# Microbiome preterm birth DREAM challenge: Crowdsourcing machine learning approaches to advance preterm birth research

**DOI:** 10.1016/j.xcrm.2023.101350

**Published:** 2023-12-21

**Authors:** Jonathan L. Golob, Tomiko T. Oskotsky, Alice S. Tang, Alennie Roldan, Verena Chung, Connie W.Y. Ha, Ronald J. Wong, Kaitlin J. Flynn, Antonio Parraga-Leo, Camilla Wibrand, Samuel S. Minot, Boris Oskotsky, Gaia Andreoletti, Idit Kosti, Julie Bletz, Amber Nelson, Jifan Gao, Zhoujingpeng Wei, Guanhua Chen, Zheng-Zheng Tang, Pierfrancesco Novielli, Donato Romano, Ester Pantaleo, Nicola Amoroso, Alfonso Monaco, Mirco Vacca, Maria De Angelis, Roberto Bellotti, Sabina Tangaro, Abigail Kuntzleman, Isaac Bigcraft, Stephen Techtmann, Daehun Bae, Eunyoung Kim, Jongbum Jeon, Soobok Joe, The Preterm Birth DREAM Community, Kevin R. Theis, Sherrianne Ng, Yun S. Lee, Patricia Diaz-Gimeno, Phillip R. Bennett, David A. MacIntyre, Gustavo Stolovitzky, Susan V. Lynch, Jake Albrecht, Nardhy Gomez-Lopez, Roberto Romero, David K. Stevenson, Nima Aghaeepour, Adi L. Tarca, James C. Costello, Marina Sirota

**Affiliations:** 1 Division of Infectious Disease, Department of Internal Medicine, University of Michigan, Ann Arbor, MI, USA; 2 March of Dimes Prematurity Research Center at the University of California San Francisco, San Francisco, CA, USA; 3 Bakar Computational Health Sciences Institute, University of California San Francisco, San Francisco, CA, USA; 4 Department of Pediatrics, University of California San Francisco, San Francisco, CA, USA; 5 Sage Bionetworks, Seattle, WA, USA; 6 Benioff Center for Microbiome Medicine, Department of Medicine, University of California, San Francisco, San Francisco, CA, USA; 7 Department of Pediatrics, Stanford University School of Medicine, Stanford, CA, USA; 8 March of Dimes Prematurity Research Center at Stanford University, Stanford, CA, USA; 9 Data Core, Shared Resources, Fred Hutchinson Cancer Center, Seattle, WA, USA; 10 Department of Biostatistics and Medical Informatics, University of Wisconsin-Madison, Madison, WI, USA; 11 Dipartimento di Scienze del Suolo, della Pianta e degli Alimenti, Università degli Studi di Bari Aldo Moro, Bari, Italy; 12 Istituto Nazionale di Fisica Nucleare, Sezione di Bari, Bari, Italy; 13 Dipartimento Interateneo di Fisica “M, Merlin”, Università degli Studi di Bari Aldo Moro, Bari, Italy; 14 Dipartimento di Farmacia – Scienze del Farmaco, Università degli Studi di Bari Aldo Moro, Bari, Italy; 15 Department of Biological Sciences, Michigan Technological University, Houghton, MI, USA; 16 School of Electrical Engineering and Computer Science, Gwangju Institute of Science and Technology (GIST), Gwangju, Republic of Korea; 17 Korea Bioinformation Center (KOBIC), Korea Research Institute of Bioscience and Biotechnology (KRIBB), Daejeon, Republic of Korea; 18 Department of Biochemistry, Microbiology and Immunology, Wayne State University, Detroit, MI, USA; 19 Imperial College Parturition Research Group, Division of the Institute of Reproductive and Developmental Biology, Imperial College London, London, UK; 20 March of Dimes Prematurity Research Centre at Imperial College London, London, UK; 21 Center for Computational Biology and Bioinformatics, Columbia University, New York, NY, USA; 22 Thomas J. Watson Research Center, IBM, Yorktown Heights, NY, USA; 23 Sema4, Stamford, CT, USA; 24 Division of Gastroenterology, Department of Medicine, University of California, San Francisco, San Francisco, CA, USA; 25 Perinatology Research Branch, Division of Obstetrics and Maternal-Fetal Medicine, Division of Intramural Research, Eunice Kennedy Shriver National Institute of Child Health and Human Development, National Institutes of Health, US Department of Health and Human Services, Detroit, MI, USA; 26 Department of Obstetrics and Gynecology, University of Michigan, Ann Arbor, MI, USA; 27 Department of Epidemiology and Biostatistics, Michigan State University, East Lansing, MI, USA; 28 Center for Molecular Medicine and Genetics, Wayne State University, Detroit, MI, USA; 29 Detroit Medical Center, Detroit, MI, USA; 30 Department of Obstetrics and Gynecology, Florida International University, Miami, FL, USA; 31 Center for Academic Medicine, Stanford University School of Medicine, Stanford, CA, USA; 32 Department of Anesthesiology, Perioperative, and Pain Medicine, Stanford University School of Medicine, Stanford, CA, USA; 33 Department of Biomedical Data Sciences, Stanford University School of Medicine, Stanford, CA, USA; 34 Department of Obstetrics and Gynecology, Wayne State University School of Medicine, Detroit, MI, USA; 35 Department of Computer Science, Wayne State University College of Engineering, Detroit, MI, USA; 36 Department of Pharmacology, University of Colorado Anschutz Medical Campus, Aurora, CO, USA; 37 Department of Pediatrics, Obstetrics and Gynaecology, Universidad de Valencia, Valencia, Spain; 38 IVIRMA Global Research Alliance, IVI Foundation, Instituto de Investigación Sanitaria La Fe (IIS La Fe), Valencia, Spain

**Keywords:** preterm birth, vaginal microbiome, machine learning, predictive modeling, crowdsourced, microbiome, 16S harmonization, DREAM challenge

## Abstract

Every year, 11% of infants are born preterm with significant health consequences, with the vaginal microbiome a risk factor for preterm birth. We crowdsource models to predict (1) preterm birth (PTB; <37 weeks) or (2) early preterm birth (ePTB; <32 weeks) from 9 vaginal microbiome studies representing 3,578 samples from 1,268 pregnant individuals, aggregated from public raw data via phylogenetic harmonization. The predictive models are validated on two independent unpublished datasets representing 331 samples from 148 pregnant individuals. The top-performing models (among 148 and 121 submissions from 318 teams) achieve area under the receiver operator characteristic (AUROC) curve scores of 0.69 and 0.87 predicting PTB and ePTB, respectively. Alpha diversity, VALENCIA community state types, and composition are important features in the top-performing models, most of which are tree-based methods. This work is a model for translation of microbiome data into clinically relevant predictive models and to better understand preterm birth.

## Highlights

• DREAM challenge participants predicted preterm birth from vaginal microbiome data
• Top machine learning model submissions achieved AUROC scores up to 0.87
• Alpha diversity, VALENCIA community state types, and composition were important features

## Graphical abstract

**Figure:**
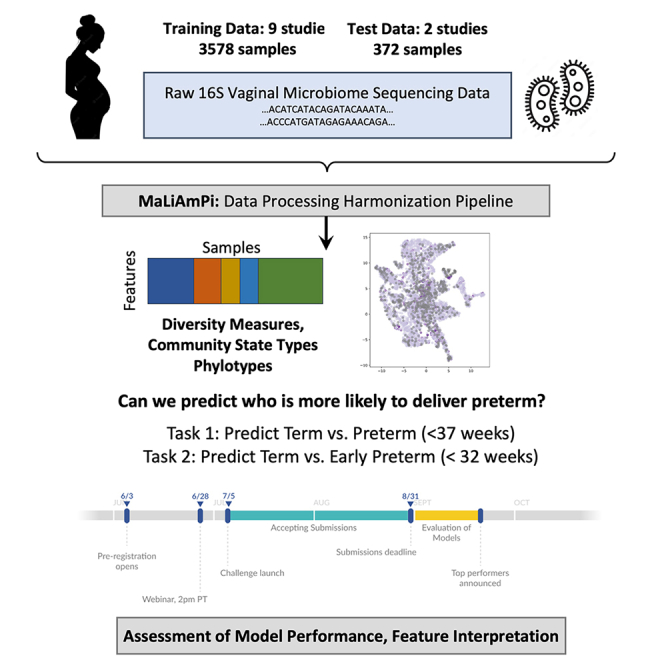

## Teaser

Golob et al. present work from their international challenge to predict preterm birth from vaginal microbiome. Top-ranking teams’ machine learning models achieved acceptable to excellent prediction. This work serves as the foundation for subsequent efforts to translate predictive tests into clinical practice and to better understand and prevent preterm birth.

## Introduction

Preterm birth (PTB) is the leading cause of infant morbidity and mortality worldwide. Globally, every year approximately 11% of infants are born preterm, defined as birth prior to 37 weeks of gestation, totaling nearly 15 million births.^1^ In addition to the emotional and financial toll on families, PTBs result in higher rates of neonatal death, nearly 1 million deaths each year, and long-term health consequences for some children.^2^ Infants born preterm are at risk for a variety of adverse outcomes, such as respiratory illnesses, cerebral palsy, infections, and blindness, with infants born early preterm (i.e., before 32 weeks) at increased risk of these conditions.^3^ Thus, the ability to accurately identify women at risk for PTB is a first step in the development and implementation of treatment and prevention strategies. Currently, available treatments for pregnant individuals at risk of preterm delivery include corticosteroids for fetal maturation and magnesium sulfate provided prior to 32 weeks to prevent cerebral palsy.^2^ Progesterone supplementation may also be administered as early as the second trimester to reduce the risk of PTB.^4^

There are several known factors associated with PTB, including history of PTB, a short cervix, extremes of maternal age and body mass index (BMI), low socio-economic status, smoking, and genetic polymorphisms.^5^^,^^6^^,^^7^^,^^8^^,^^9^^,^^10^^,^^11^ Nevertheless, there is a need for additional clinical tools that enable the early and reliable assessment of the risk of PTB for an individual^12^^,^^13^ with quantitative rigor. Machine learning (ML) modeling has demonstrated potential to aid in the determination of individuals at risk of conditions and diseases across medical domains.^14^^,^^15^^,^^16^ By applying ML methods to large amounts of heterogeneous data, patterns in data can be discerned that would be otherwise difficult for humans to distinguish. Moreover, deducing which features contribute most to the predictive performance of an ML model allows for the identification of biomarkers that can be important for a condition or disease. There are a variety of ML algorithms that can be used individually or combined into an ensemble approach to improve prediction performance. After ML modeling has been applied to and optimized on a training dataset, the model is then ideally tested on an independent dataset to assess how well the model is able to generalize to data it has never seen before.^17^ The validation on independent data is a critical step to guard against overfitting and hence optimistically biased accuracy estimates. In the past several decades, applications of ML approaches to various types of clinical, molecular, and other data have been explored to predict complications of pregnancy including PTB.^18^^,^^19^^,^^20^^,^^21^^,^^22^^,^^23^ The results of these works to date demonstrate that the prediction of PTB from varied data types including metabolites in amniotic fluid and maternal blood and urine, ultrasound images, and electronic health records appears to be feasible to a certain extent. In 2019, a DREAM (Dialogue for Reverse Engineering Assessments and Methods) Challenge was organized to harness the power of crowdsourcing and engage the computational biology community to develop and apply ML models to maternal blood multi-omics data for the determination of gestational age at time of blood draw and prediction of spontaneous PTB.^24^

There is some indication that the vaginal microbiome is associated with adverse pregnancy outcomes, specifically PTB. Previous studies have shown that there are significant differences between the vaginal microbiome of patients who deliver at term and those who deliver prematurely. Vaginal microbiomes with increased diversity as well as communities where *Lactobacillus* is not dominant were more frequent in patients with PTB.^25^^,^^26^^,^^27^ Therefore, the vaginal microbiome is a tempting source of data to use for predictive modeling of PTB. However, there are significant biological and technical challenges to using microbiome data for predictive modeling. Biologically, human-associated microbiomes (including the vaginal microbiome) are incredibly variable—with any two individuals typically sharing less than half of microbes at the sequence-variant level of resolution.^28^ Thus, microbiome data, particularly compositional microbiome data, are both highly dimensional and sparse. These microbiome data attributes contribute to a substantial risk of model overfitting. Meta-analysis as well as rigorous evaluation of models on independent validation data is a robust approach to contend with these biological challenges with microbiome data. However, there are significant technical challenges in aggregating and combining microbiome data across studies; therefore, there have been few studies taking on this task.^29^^,^^30^^,^^31^ In previous work, we have shown that by aggregating microbiome data across several studies, we can gain significant statistical power, reproduce that higher alpha diversity is associated with PTB, especially in the first trimester of pregnancy, and identify several microbial associations.^32^

We hypothesized that applying advanced computational and ML techniques to aggregated microbiome data across many diverse studies could be used successfully for the identification of women at risk of delivering preterm. While ML approaches have been applied to the vaginal microbiome, most have involved a single dataset with limited sample size.^33^^,^^34^^,^^35^ One recent work explored the application of ML to 12 vaginal microbiome datasets to predict PTB; they leveraged public data extensively to ensure that their findings were robust across studies, but their work did not include an independent validation dataset.^29^ The prediction quality of their random forest model had an area under the receiver operator characteristic (AUROC) curve score from 0.28 to 0.79. New tactics for harmonizing microbiome data via phylogenetic placement^36^^,^^37^^,^^38^^,^^39^ pointed to a possible technical basis for combining multiple studies into a training set suitable for more rigorous predictive modeling. Further, we hypothesized that crowdsourcing models that would be independently evaluated against validation data not available to the model developers could result in models less prone to overfitting and thus with better predictive quality.

Building on the groundwork laid by the 2019 Preterm Birth Transcriptome Prediction DREAM Challenge,^24^ we designed a new DREAM Challenge aimed at leveraging longitudinal microbiome data and crowdsourcing for prediction of (1) PTB or (2) early PTB. DREAM Challenges define the prediction task, supply the necessary data, and provide the infrastructure to evaluate models designed by any participating teams; they do so in an unbiased manner using a gold-standard, undisclosed validation dataset. The Challenges are international, open-science efforts to identify the best predictive models. Here, we provide the results from the Preterm Birth Microbiome Prediction Challenge, along with the top models, and insights gained from this initiative. The dockerized code for all predictive pipelines has been made available along with data used in the challenge at http://www.synapse.org/preterm_birth_microbiome. This work can serve as the foundation for subsequent endeavors to better understand the mechanisms underlying PTB and early PTB and to translate into clinical practice predictive tests to help identify women at risk of delivering preterm. Likewise, we believe this is a robust scientific approach suitable for predictive modeling of other conditions based on microbiome data.

## Results

### Overview

**Figure 1:**
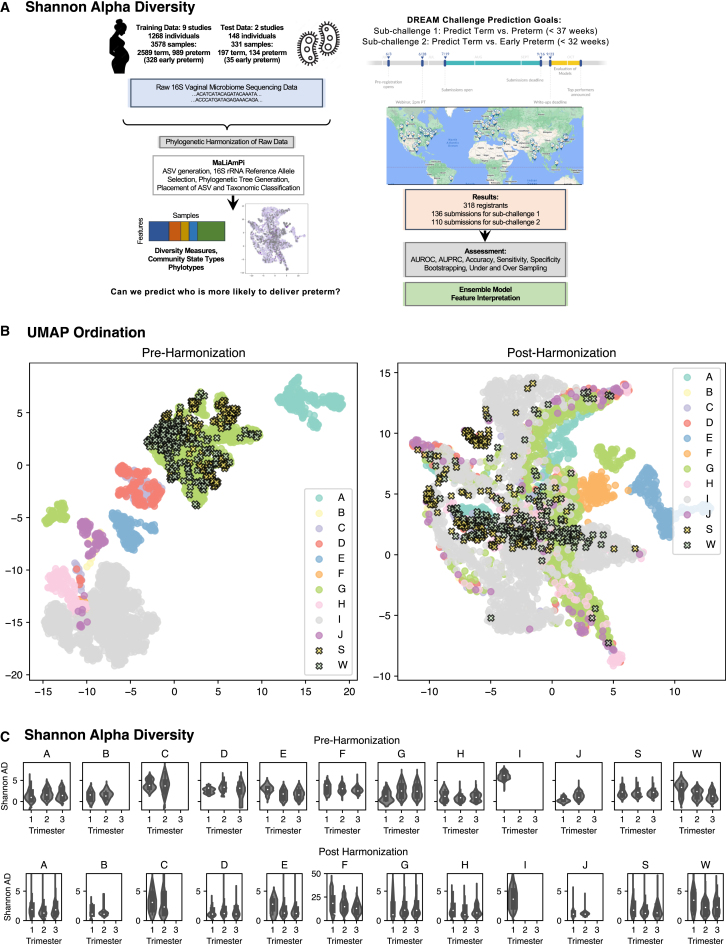
Study design and challenge overview and data harmonization (A) Left: depiction of the assembled training and test datasets, harmonization of the data, transformation into feature tables, and the outcomes posed to the participating teams. Right: two sub-challenges, the global locations of the participating teams, the number of participants per sub-challenge, assessment process, and analysis of the better-performing models. (B) Uniform manifold approximation and projection (UMAP) ordination plots of the aggregated data before (left) and after (right) harmonization where each dot represents one vaginal microbiome sample colored by study. (C) Violin plots of Shannon alpha diversity by trimester before (top) and after (bottom) harmonization stratified by study.

The overall timeline of the Microbiome PTB DREAM challenge is shown in Figure 1A. Major milestones included development and harmonization of the training data, opening of the challenge to participants, post hoc integration and harmonization of the validation data, assessment of models, and finally, evaluation of the approaches and results. We leverage data across 9 studies including over 3,500 samples and utilized crowdsourcing to identify the best predictive strategies and models for prediction of PTB. The endpoints of the challenge included PTB (delivery before 37 weeks of gestation) and early PTB (delivery before 32 weeks of gestation).

### Data aggregation and processing

The training dataset was constructed by aggregating and processing vaginal microbiome data from the public domain leveraging resources including dbGAP^40^ as well as MOD Database for Preterm Birth Research.^41^ The final dataset included data from nine studies, representing 3,578 samples from 1,268 individuals. Of these patients, 851 delivered at term and 417 preterm (before 37 weeks of gestation), including 170 whose deliveries were early preterm (before 32 weeks of gestation). Details of the nine studies that were included in the training set are shown in Table S1. Figure S1 illustrates the sampling strategies for each of the datasets, showing that some studies (like I and J) collected samples only once during gestation, while in most other studies, samples were collected multiple times during gestation from the same individual. While all of these studies focus on profiling the 16S rRNA gene, primers targeting different variable regions of the 16S rRNA gene, PCR conditions, and sequencers all varied. The combination of microbiome data from different studies, particularly those using different underlying techniques, is a challenging task that has hindered prior efforts for meta-analysis of microbiome data in a manner distinct from other forms of ‘omics data, like transcriptomics and genotyping. To emphasize that it is not biologically correct to combine technically diverse 16S-based microbiome studies at the raw sequence level, ordination based on denoised and then dereplicated amplicon sequence variant (ASV) pseudo-counts results in specimen clustering by technique, as expected when non-overlapping variable regions of the 16S rRNA gene are being amplified (Figure 1B, left). Thus, we first focused on harmonizing the data from the nine studies that comprised our training sets. This was done based on phylogenetic placement of the ASVs onto a common *de novo* maximum-likelihood phylogenetic tree comprised of full-length 16S rRNA alleles using a Nextflow-based workflow called MaLiAmPi.^42^ After processing with MaLiAmPi, we were able to overcome most of the technique-based noise and successfully harmonize the data into one cohesive feature set of compositional features, as evidenced by the integration of the studies after uniform manifold approximation and projection (UMAP) ordination, this time based on phylotype counts (Figure 1B, right). Ultimately, the true relationship between specimens is unknown. But, after harmonization, it is reassuring that each study now has specimens overlapping and broadly representing the topography after UMAP ordination, as well as t-distributed stochastic neighbor embedding (tSNE) and multidimensional scaling ordination (Figure S2). Additional dimensionality reduction plots demonstrating the successful integration of the data, colored by trimester of collection and demographic features, are presented in Figure S3.

A similar challenge arises when comparing alpha diversity (metrics of richness and/or unevenness in a microbial community) between studies, where the estimates can be affected by total reads recovered per specimen as well as the specific variable region of the 16S rRNA gene being targeted.^43^ To demonstrate this point, the Shannon alpha diversity estimates based on denoised and dereplicated ASV counts were inconsistent in range between the studies (Figure 1C, top). Alpha diversity can be estimated after phylogenetic placement of ASVs,^44^ including the estimation of Shannon and inverse-Simpson alpha diversity via Chao numbers,^45^ but cannot fully overcome all of the limitations of cross-study comparison of alpha diversity (Figure 1C, bottom). In particular, project F (pyrosequencing based) resulted in an estimated Shannon diversity an order of magnitude higher than would typically be expected. Participating teams were presented these post-harmonization results as well as raw ASV counts if they wished to rederive the alpha diversity metrics.

**Figure 2:**
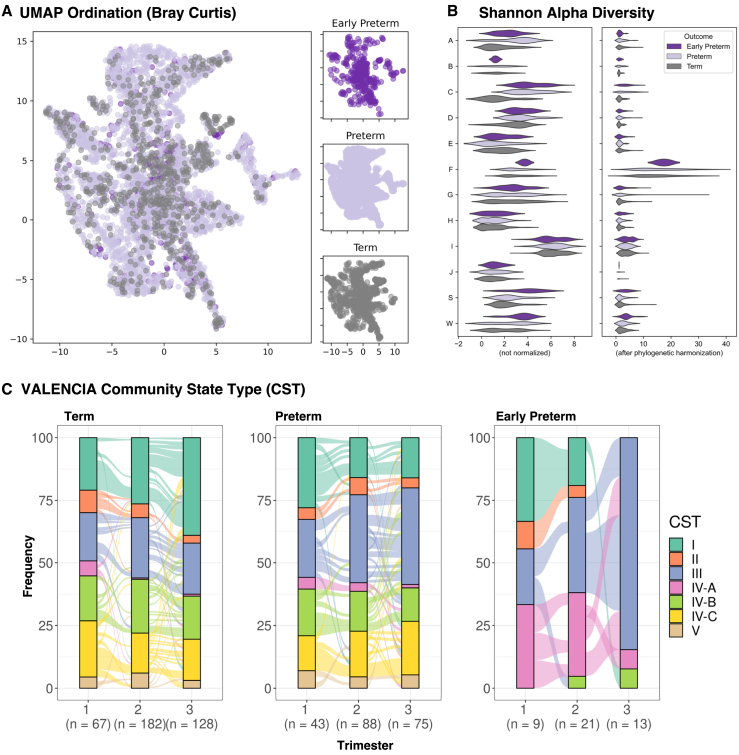
Data visualization of microbiome features by outcome (A) UMAP ordination plots of the vaginal microbiome colored by outcome. (B) Violin plot of diversity before (left) and after (right) harmonization stratified and colored by outcome. (C) Alluvial plot of community state type (CST) frequencies across time stratified by birth outcome.

The separation between samples by outcome—from term, preterm, and early preterm deliveries—is not clearly evident (Figures 2A and 2B). There are some distinct differences observed with respect to community state types (CSTs) and outcome (Figures 2C and S4). Leveraging different types of microbial features including phylotype relative abundance and diversity measures as well as CST membership provides an opportunity to apply ML techniques to these data for PTB prediction.

To build an independent test set for evaluating the models submitted by participants in this DREAM challenge, we combined an unpublished dataset from Wayne State University consisting of 159 samples across 60 individuals, among whom 40 (66.7%) had term deliveries and 20 (33.3%) had preterm deliveries, including 5 (8.3%) who had early preterm deliveries (Table 1). Most patients in this test set had three longitudinal samples. We also generated a second validation dataset that comprised 172 vaginal microbiome samples from 88 individuals, up to three samples (one sample per trimester) for each individual, with 48 individuals (54.5%) having term deliveries and 40 individuals (45.5%) having preterm deliveries, including 8 (9.1%) having early preterm deliveries. DNA extraction, V4 16S rRNA gene library preparation, and 16S rRNA gene sequencing (2 × 150 paired-end sequencing on the Illumina NextSeq platform) of these samples were performed by the UCSF Benioff Center for Microbiome Medicine, with most samples yielding over 100,000 reads (see STAR Methods for details). Figure S1 represents the week of gestation for the sample collection times for each individual from the two test datasets. These validation datasets became available only after the training dataset was generated and distributed to teams. Thus, the resultant reads had to be integrated into the same feature set as in the training data post hoc. Using MaLiAmPi, we were able to first generate the training data, preserving the features (e.g., phylotypes, alpha diversity, etc.) (Figures 2A and 2B) and further integrating the validation datasets. The generalizability of these features across studies, including new study data, has allowed us to apply the ML models to these independent validation sets and enable the use of the model on data to be generated in the future.

**Table 1:** Summary of demographics of training (A‒J) and validation (S and W) datasets

	Group	Total	Training (A–J)	Validation (S and W)
Individuals	n	1,416	1268	148
Age range, n (%)	unknown	691 (48.8)	691 (54.5)	0 (0)
	below 18	4 (0.3)	4 (0.3)	0 (0)
	18–28	304 (21.5)	227 (17.9)	77 (52.0)
	28–38	357 (25.2)	293 (23.1)	64 (43.2)
	above 38	60 (4.2)	53 (4.2)	7 (4.7)
Race, n (%)	American Indian or Alaska Native	9 (0.6)	6 (0.5)	3 (2.0)
	Asian	84 (5.9)	81 (6.4)	3 (2.0)
	Black or African American	827 (58.4)	759 (59.9)	68 (45.9)
	Native Hawaiian or Other Pacific Islander	7 (0.5)	3 (0.2)	4 (2.7)
	White	422 (29.8)	360 (28.4)	62 (41.9)
	unknown	71 (5.0)	63 (5)	8 (5.4)
Ethnicity, n (%)	Hispanic or Latino	50 (3.5)	8 (0.6)	42 (28.4)
	unknown	1,261 (89.1)	1,260 (99.4)	1 (0.7)
Delivery, n (%)	term	939 (66.3)	851 (67.1)	88 (59.5)
	preterm	477 (33.7)	417 (32.9)	60 (40.5)
	early preterm	183 (12.9)	170 (13.4)	13 (8.8)

### The DREAM challenge results

The Preterm Birth Microbiome Prediction DREAM Challenge launched on July 5, 2022 (Figure 1A), and closed on September 16, 2022. There were two sub-challenges: sub-challenge 1, prediction of PTB (before 37 weeks of gestation), and sub-challenge 2, prediction of early PTB (before 32 weeks of gestation). The validation dataset for this second sub-challenge included only data from samples collected no later than 28 weeks of gestation (to reduce trivial predictions based upon later-in-gestation specimens being available from a pregnancy). A baseline “organizers” random-forest-based model was developed with the training data to provide participants an example, inclusive of packing of the model within a docker container. Performance metrics that were used to evaluate the prediction models submitted by the teams include area under the receiver operator characteristic (AUROC) curve, area under the precision-recall (AUPR) curve, accuracy, sensitivity, specificity, and Matthews correlation coefficient (MCC). All values were determined on bootstrapped validation data, with the mean bootstrapped value used to evaluate the model. The primary scoring metric was set at the onset to be AUROC, followed by AUPR to break ties.

**Figure 3:**
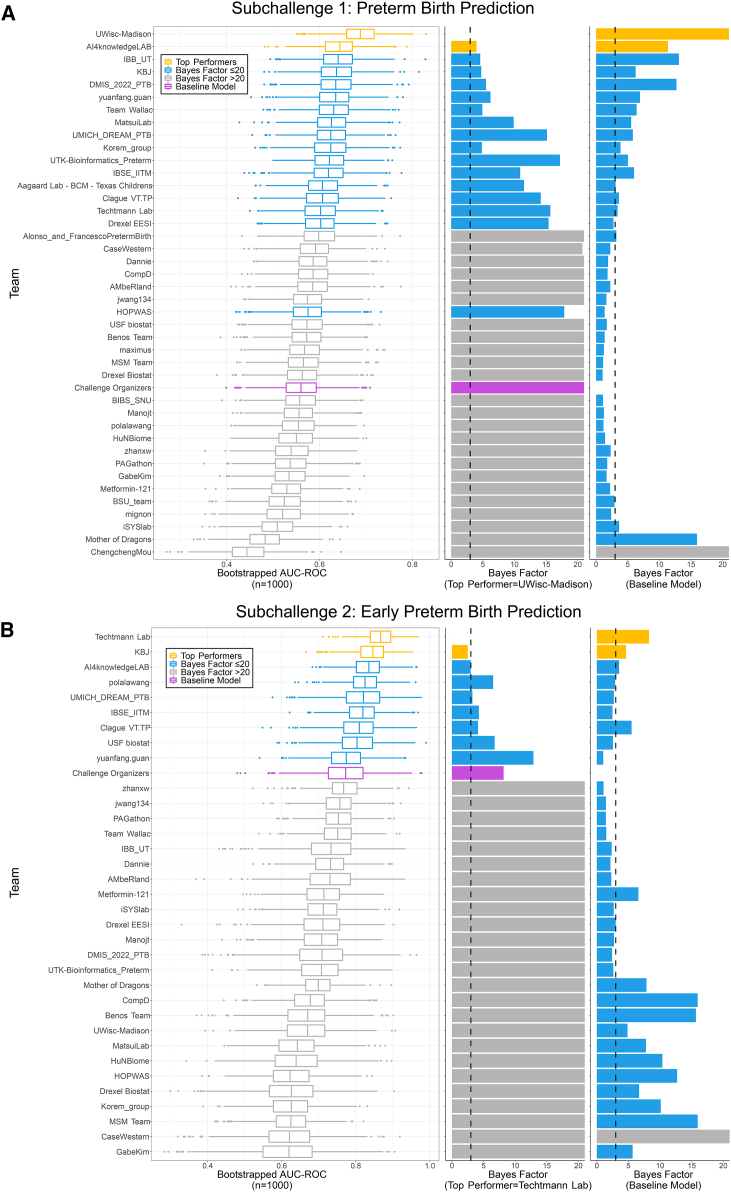
Prediction accuracy of models against sequestered validation data from two independent studies not available to modeling teams Bootstrapped area under the receiver operator characteristic (AUROC) curves and Bayes factors for (A) sub-challenge 1 and (B) sub-challenge 2 of the best-performing model of each team for each sub-challenge and the organizer’s baseline model (purple) against bootstrapped data (n = 1,000) with replacement from the two validation studies harmonized post hoc into the same feature sets. Bootstrapping was done by pregnancy, not specimen. Left column: box-and-whisker plots of the bootstrapped AUROC values; middle column: the Bayes factors when compared to the top-performing model; right column: Bayes factors when comparing against the organizer’s model. Yellow represents the two best-performing models for each sub-challenge. Blue represents models with a Bayes factor ≤20 when compared to the top-performing model.

There were 318 participants from all over the world with 136 and 110 submissions for sub-challenges 1 and 2, respectively. The prediction models with top-ranking submissions achieved mean bootstrapped AUROC scores of 0.688 and 0.868, respectively, for the 2 sub-challenges (Figure 3; Tables S2 and S3). Several techniques were carried out in order to ensure the robustness of the resulting rankings including test set label inversion, bootstrapping, oversampling, and undersampling (see STAR Methods). The results are shown in Figure S5 (sub-challenge 1) and Figure S6 (sub-challenge 2).

A few patterns emerged in the best-performing predictive models for sub-challenge 1 (Table S4) and sub-challenge 2 (Table S5). Nearly all of the models used tree-based approaches (typically implemented as part of the python Scikit Learn^46^ package), such as random forest and relatives. A few models used regression approaches with inclusion of gestational age at sampling (with feature pruning and clustering) or neural networks. All of these modeling approaches are notable for their aggressive pruning or consolidation of features well-suited for handling both sparse and highly dimensional data. Therefore, avoiding overfitting the training data was a shared and likely essential attribute of the best-performing models.

#### Predictive features

**Figure 4:**
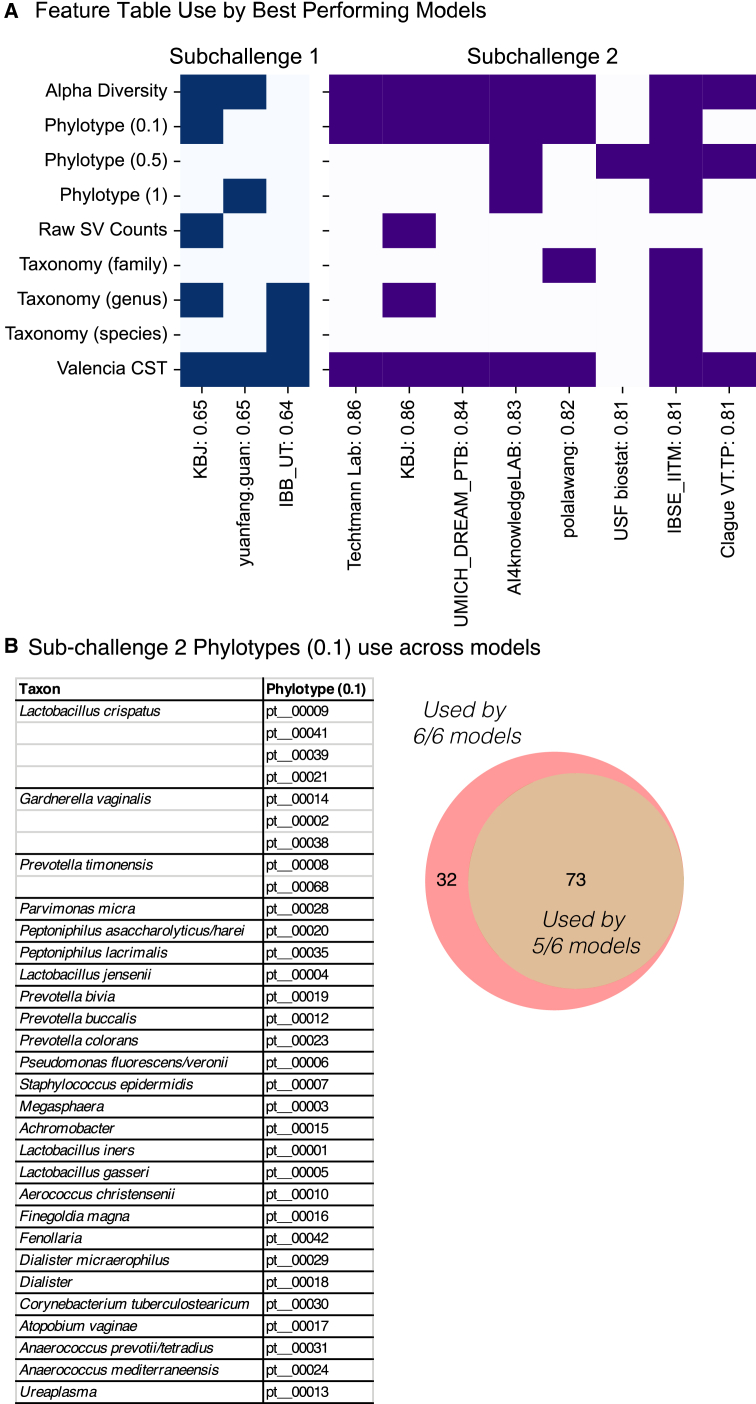
Feature sets and individual compositional features used by top-performing models Top-performing models here are defined a bootstrapped area under receiver operator curve greater than 0.64 or 0.8, respectively, for sub-challenge 1 or 2, further limited to models that could make a prediction in less than 10 s on a twelve-core AMD Ryzen 3900X processor. (A) Feature tables used by the top-performing models for sub-challenge 1 (left) and sub-challenge 2 (right) to make their predictions of preterm birth and early preterm birth, respectively. Filled in blocks indicate that this feature table (by row) was used by a given model (columns) to make the prediction. Unfilled blocks are for feature tables that, when randomized, did not affect the prediction. (B) For the six sub-challenge 2 models evaluated by feature permutation that also made use of phylotypes at 0.1 distance, 32 of the phylotypes were used by all 6 models and 73 were used by 5 of the six models (right Venn diagram). 32 phylotypes used by all six models are grouped by the closest species (left) for that phylotype.

We identified microbiome features that the best-performing models (as judged by mean bootstrapped AUROC cutoffs of 0.64 and 0.8 for sub-challenges 1 and 2, one model per team and limited to models that could make a prediction in a tractable time) relied upon to make their predictions, resulting in the evaluation of three models for sub-challenge 1 and eight models for sub-challenge 2. We used feature permutation as a means of empirically identifying the feature tables and, in turn, specific features that the models depended upon for their predictions, with an emphasis on identifying features used by multiple independently developed models. Teams were provided multiple feature tables but had no requirement to use any specific table for making predictions. These included a table of alpha diversity metrics; composition via phylotypes at three distinct resolutions; composition via taxonomy at the species, genus, or family level; and VALENCIA CSTs. Feature permutation at the table level revealed broad use of alpha diversity metrics (2 out of 3 and 7 out of 8 for sub-challenges 1 and 2 respectively), VALENCIA CSTs (3 out of 3 and 7 out of 8 for sub-challenges 1 and 2, respectively), and composition via phylotypes (2 out of 3 and 8 out of 8 for sub-challenges 1 and 2, respectively). In contrast, composition via taxonomy was used by 3 of the 8 better-performing models for sub-challenge 2 (Figure 4A).

We next identified specific features used by the top-performing predictive models in both sub-challenges via feature permutation, narrowing in on alpha diversity and compositional features used consistently by the better-performing models (Tables S6 and S7). For compositional features (phylotypes or taxons), we further considered individual features found in at least 10% of the specimens in the training set (to reduce the computational load). There was notable convergence between the features used by the models to make their predictions. For sub-challenge 1, the two models that made use of alpha diversity made use of the same seven metrics. For the two models that made use of genus-level compositional data, 68 genera were used by both models. For sub-challenge 2, all seven models that used alpha diversity had predictions that were sensitive to rooted phylogenetic diversity. Of the six models that made use of the phylotypes binned at 0.1 distance (of which 105 of the phylotypes were above 10% density), 32 of these phylotypes were used by all six models for their prediction (Figure 4B). As expected based on our parallel evaluation of phylotypes relative to taxonomy,^47^ a given species is frequently split among multiple phylotypes when binned at a distance of 0.1. Four phylotypes like *Lactobacillus crispatus*, three phylotypes like *Garnerella vaginalis*, and two *Prevotella timonensis*-like phylotypes were used by all six models when making predictions. One model for sub-challenge 2 (USF biostat) was able to make quite an accurate prediction while only making use of phylotypes binned at 0.5 distance—with comparable prediction performance to models making use of a much broader set of feature tables (Figure 4B).

We performed univariate correlation with features used by at least one of the better-performing sub-challenge 1 or sub-challenge 2 models with PTB or early PTB (ePTB), respectively. For each alpha diversity metric and VALECINA CST, we used generalized linear modeling to estimate the univariate correlation. For taxons and phylotypes, we chose detected/not detected to account for these features being sparse (detected only in a small minority of microbiota). To account for repeated sampling, we averaged by pregnancy across each trimester. Overall, as we expected, the univariate analysis revealed complex and trimester-dependent relationships with PTB and ePTB (Figure S6 and S7) that the ML models were able to overcome.

#### Sensitivity analysis on gestational age at sampling

To ensure that the best-performing models were not overly reliant upon the gestational week of collection of specimens, we performed a sensitivity analysis—removing gestational age at sampling or permuting gestational age values (Table 2). Model performance was only modestly affected by removing model access to the gestational age of collection, indicating that the predictions were primarily based on other attributes.

**Table 2:** Sensitivity analysis removing gestational age as a feature for sub-challenge 1 and sub-challenge 2

Team	AUROC	AUPRC	Accuracy	Sensitivity	Specificity	MCC
**Sub-challenge 1**						

AI4KnowledgeLAB	0.599	0.448	0.608	0.367	0.773	0.152
UWisc-Madison	0.690	0.560	0.689	0.417	0.875	0.334

**Sub-challenge 2**						

KBJ	0.820	0.236	0.781	0.692	0.790	0.316
Techtmann lab	0.844	0.343	0.911	0.000	1.000	–

#### Post-challenge ensemble models

**Figure 5:**
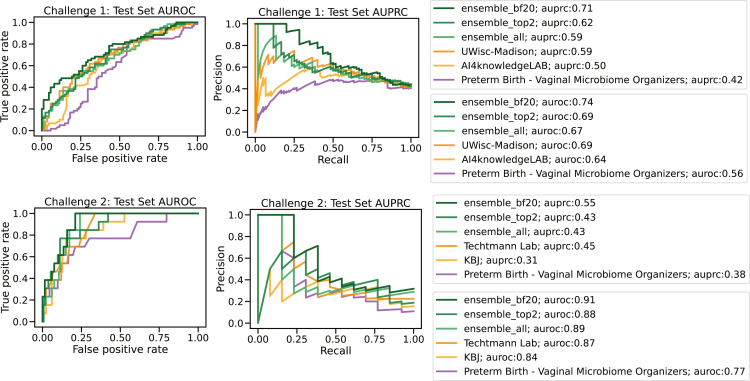
Ensemble model results For (A) sub-challenge 1 and (B) sub-challenge 2, the AUROC (left) curve and area under the precision-recall curve (AUPRC; right) of three ensemble models (“ensemble_top2”: top two best-performing models, “ensemble_top2”: models with Bayes factor less than 20, and “ensemble_all”: all models), as well as first place, second place, and baseline models, colored by model.

Several ensemble models were created, combining results of (1) the winning teams, (2) the teams with Bayes factor <20 (Figure 3), and (3) all participants across the two sub-challenges (Figure 5). The underlying models (as noted above) make use of many of the same microbiome features. Still, the ensemble models were evaluated against the two validation studies unavailable to the model developers to avoid artificially improved scores due to overfitting. An improvement in performance was observed across the board, with the ensemble models of Bayes factor <20 performing the best, with AUROC values of 0.74 and 0.91, respectively, for sub-challenges 1 and 2.

## Discussion

PTB, particularly ePTB (before 32 weeks of gestation), remains a potentially devastating outcome of pregnancy. Without a clear way of identifying pregnancies at risk for PTB, it remains difficult to target interventions or clinical trials. The microbiome has been extensively correlated in single-center studies with the risk for PTB, fueling the idea of using the vaginal microbiome to build rigorous, generalizable, and robust predictive models to identify pregnancies at risk for PTB. Both a strength and a challenge arise from the biological and technical diversity of these studies. Specifically, combining data from different microbiome studies into a predictive, stable, and generalizable set of features for the rigorous evaluation of predictive models against independent validation datasets and their eventual use with vaginal microbiome data from individual pregnancies clinically is technically non-trivial. In this study, we leveraged data from 9 independent studies of the vaginal microbiome during pregnancy. The data were aggregated from public domain sources including dbGAP and the MOD Database for Preterm Birth Research. The final training dataset included data from 3,578 samples across 1,268 individuals, with 851 individuals delivering at term and 417 delivering preterm, including 170 early preterm deliveries. We applied a scientific and technical schema, implemented in a software workflow, MaLiAmPi, and building upon previous efforts using phylogenetic placement,^36^^,^^37^^,^^38^^,^^39^ for harmonizing microbiome data at the sequence level, even when generated with different underlying primers and sequencing platforms, to transform the raw data into a stable and generalizable set of features suitable for predictive modeling. This schema also allowed the post hoc integration of microbiome data from two independent validation sets (that were unavailable at the time of the release of the training set) into the same set of features: an unpublished dataset from Wayne State University/Perinatology Research Branch and a second validation dataset generated by UCSF from samples provided by Stanford University. Crowdsourced predictive models were developed by 318 teams based on the training feature set and evaluated against the independent validation data within the same schema of features. Multiple teams were able to generate predictive models for both PTB and ePTB, with the models predicting the latter particularly robustly.

We believe that these models are the nucleus of a robust and clinically relevant test to identify pregnancies at the highest risk for ePTB, a long-standing need in clinical obstetrics.^48^ If developed further into a clinically orderable test either as a laboratory-developed test or *in vitro* diagnostic,^49^ robust and accurate vaginal-microbiome-based identification of pregnancies at risk for preterm labor would allow for marshaling of familial and institutional resources to support these vulnerable pregnancies. Further, this vaginal-microbiome-based test identifying pregnancies at risk for preterm labor could facilitate practical interventional preventative trials by enriching the study population to those at the highest risk for PTB. From a research perspective, contrasting the predictions from vaginal-microbiome-based predictive models with those from electronic medical records, serum biomarkers, or other data sources could help us better understand the underlying causal relationships (host, microbiome, or both) contributing to the risk of preterm labor. One strength here from the crowdsourcing approach is the availability of multiple independently developed predictive models with nearly identical predictive performances. This both bolsters the confidence in the underlying ability to accurately identify pregnancies at risk for preterm labor with vaginal microbiome data as well as success with eventual clinical translation of these models.

Both the training and validation data were heterologous not just in their techniques but also in their study populations and sampling schedules (Figure S1). Further, modeling teams were not provided a guaranteed sampling schedule (e.g., “three samples from the second trimester”) for the validation data and instead had to have some method to be flexible enough to handle an unknown degree of sampling of a pregnancy. Teams were encouraged to carefully consider this attribute of the data and in turn employed a variety of strategies that included over- and undersampling, ensemble modeling, and masking of data from some of the training studies. Approaches to address repeated sampling of a pregnancy, evaluate if there is a benefit (in prediction accuracy) from repeated sampling, and make maximal use of the available microbiome data are all exciting opportunities for future studies. Likewise, while we employed cutting-edge approaches for the harmonization of technically diverse 16S rRNA data, the harmonization was imperfect. This can be most clearly seen in the alpha diversity estimates (Figure 3B). The ongoing challenges of harmonizing technically diverse microbiome data was another barrier that model developers had to overcome. The result is a mix of limitations and strengths of this study. Specifically, multiple independent modeling teams were able to develop models with an ability to generalize their accurate predictions across technique and study populations. Still, there remains a need to validate the predictive performance in prospective studies with better-defined inclusion criteria and sampling schedules.

We noted that the best-performing predictive models all employed some type of feature pruning and selection, typically within the broad family of random-forest-like models. Given the sparseness of microbiome data and the plethora of features that can be detected, it is not surprising that modeling techniques more resilient to overfitting and better able to home in on the most important features performed better. This risk of overfitting also speaks more broadly to the value of validating microbiome associations and predictive models on independent datasets; even with a large training dataset consisting of multiple studies, teams often needed to adjust their models to reduce the risk of overfitting to perform well against the validation data.

Both taxon (species, genus, and family level) and phylotype counts were provided to teams to represent the composition of the microbiota, and the relative use of the taxonomy-based and taxonomy-free phylotypes by models gives us some insights into the relative strengths and weaknesses of the two approaches. It is notable that the taxonomy-independent phylotypes were used by a majority of the better-performing models. Taxonomy-based features were overall a challenge for participants, as there was poor overlap between studies at the taxonomic level, particularly for the less abundant or well-characterized organisms. This fits with the consensus that taxonomic assignment, particularly at the species and genus levels, is fickle, with many sequences overconfidently assigned incorrect taxonomy even when a best effort is made to correctly identify the microbes.^50^ This required teams that relied upon taxonomy to preprocess the taxonomic feature tables. In contrast, the taxonomy-independent phylotypes were more consistent between studies (over 99% of the read mass in the validation studies was assignable to a phylotype found in the training studies) and thus easier to directly input into a model. Another observation was that phylotypes were better able to capture nuances within genetically diverse taxons like *G. vaginalis* and *L. crispatus*, particularly at the finer-grained phylogenetic distance of 0.1 for binning (Figure 4B).

This study focused on prediction rather than associations or the pathophysiologic mechanisms of the vaginal microbiome and PTB. We believe that prediction is independently clinically and scientifically valuable, including acting as a means of better informing patients and providers, supporting future clinical trials and mechanistic studies focused on the subset of pregnancies at highest risk of PTB. The training dataset itself, inclusive of the stable and generalizable features, is a resource for future studies of the vaginal microbiome during pregnancy. This training set, and more importantly the stable set of features, is a possible means of avoiding a challenge in the microbiome literature, where each study reports on a slightly different set of features. Future studies can leverage this large, geographically diverse, and strictly formatted dataset to leverage and validate their findings.

A finding in our study is that more severe cases that involve early delivery were easier to predict from vaginal microbiome data than all PTB cases. This result was consistent for multiple independent modeling teams, including teams who tackled both sub-challenges, with sub-challenge 2 (predicting ePTB) models generating better predictions (as judged by our metrics, including AUROC). In ePTB, the frequency of intra-amniotic infection caused by bacteria ascending from the vagina may be higher.^56^ Further study is needed, but we believe that this could suggest that the vaginal microbiome has a stronger causal contribution to ePTB. It is both a strength and a weakness that the PTB outcomes in the training and validation studies are heterologous: spontaneous, medically indicated, etc. For the publicly available training data, these details were frequently missing. This both bolsters the generalizability of the predictions (not restricted to just the subset of spontaneous PTB) but is a limit to interpreting the results from a mechanistic or associative perspective.

Through feature permutation combined with multiple independently developed highly predictive models, we were able to identify multiple organisms, CSTs, and community structures that associate with the risk for PTB, opening the door for future studies into the underlying biology and pathophysiology of these associations, as well as more precise and effective intervention upon the vaginal microbiome during pregnancy to reduce the risk of PTB. In particular, while non-dominance of *Lactobacillus* in vaginal microbiome communities has previously been associated with PTB,^25^^,^^26^^,^^27^ there seems to be physiologically relevant species- and strain-level variability within the *Lactobacillus* and *Gardnerella* genera across pregnancy trimesters that deserves further exploration and indicates a potential role for intra-niche competition in the vaginal microbiome during pregnancy and the risk for ePTB. This may be particularly relevant to less well-resourced settings.

This work serves as the basis for several potential follow-up studies. To improve the performance of the models, additional data such as demographics, clinical data, environmental data, or data from other body sites could be incorporated into the models. To better understand the mechanisms underlying PTB and ePTB, further *in vitro* and *in vivo* validation of individual microbial features identified by the models can point to the underlying molecular mechanisms of human parturition. Studying how to in turn modulate the microbes can result in therapeutic hypotheses. Once the models have been validated and optimized, the next logical step is to translate them into clinical practice to help identify women at risk of PTB and to develop appropriate interventions to prevent PTB.

We believe this represents a genuine advancement in our ability to identify pregnancies at risk for ePTB. Given that these models rely upon a generalizable set of features that can accommodate post hoc data from individual pregnancies, these predictive models are “shovel ready” for use in clinical trials and for exploration of their potential role in the clinical care of pregnancies. Further, we believe that this scientific and technical schema could be suitable for building microbiome-based predictive models for other microbiome-related conditions.

### Limitations of the study

The study has several limitations that should be considered when interpreting the results. The study is based on publicly available data, which might not have full clinical or demographic annotations of the samples in the meta-data. In particular, the spontaneous nature of PTB could not be ascertained for all patients in the training set, and differentiating between spontaneous preterm labor and delivery and preterm prelabor rupture of the membranes was not feasible. Recent work suggests that this latter phenotype is more likely associated with the microbiome.^51^^,^^52^ While the sample size of the study is considerable, with 3,578 samples across 1,268 individuals, it may not be representative of the entire population of pregnant individuals from around the world. We only considered binary outcomes (term vs. preterm delivery) and did not take into account other important outcomes such as low birth weight or neonatal morbidity. The study is a computational challenge, so the results of the models are only as good as the data that they are trained on, and the limitations of the data may be reflected in the results. These include some loss of precision when harmonizing data from different studies using different underlying techniques and targeting different variable regions of the 16S rRNA gene. The validation studies, while independent and not available to the modeling teams, were small studies, with 148 pregnancies total, and were not peer reviewed prior to their use. Our use of bootstrapping, undersampling, and oversampling does not fully overcome the need for further prospective validation in future studies, inclusive of lower-resource settings and geographic sites. Finally, we only used data from the vaginal microbiome, which may not fully reflect the overall health of the pregnant individuals; other factors such as genetics, host response, lifestyle, or environment may also play a significant role in parturition timing.

This study was based on data generated from amplification of variable regions of the 16S rRNA gene. This is a venerable technique and provided us with a large set of already-analyzed sequences from which we could build our training set. The ability to harmonize 16S rRNA gene variable-region-based microbiome data from technically diverse studies is improved with recent technical advances but is still limited, as evidenced by our findings, such as alpha diversity estimates remaining affected by a study’s technical design (Figure 3B). Whole-genome shotgun sequencing can capture a broader swath of organisms, provide more functional insights, and better resolve the composition of a community, at the cost of significant increases in sequencing depth and computational resources needed. There are some relatively poorly characterized technical challenges when combining data from technically diverse whole-genome shotgun studies that need to be addressed.^53^ Likewise, there are many other forms of information about pregnancy, including electronic medical record data, host transcriptomics, host epigenomics, etc., that can be derived. Future studies can and should explore the predictive utility of these other forms of data, as well as consider if there is a benefit to multi-omics integration for predictive accuracy.

## STAR★Methods

### Key resources table

**Table:** 

REAGENT or RESOURCE	SOURCE	IDENTIFIER
**Deposited data**		

Exploring the human maternal microbiome and its contribution to preterm birth	Sirota et al.^41^	March of Dimes Prematurity Database SDY465; doi.org://10.21430/M3D491LGDT
The vaginal microbiota of pregnant women who subsequently have spontaneous preterm labor and delivery and those with a normal delivery at term	Romero et al.^54^	NIH SRA PRJNA242473
Vaginal Microbiome of Pregnancy	Fettweis et al.^55^	NIH SRA PRJNA294119
Replication and Refinement of a Vaginal Microbial Signature of Preterm Birth	Callahan et al.^27^	NIH SRA PRJNA393472
Multi Omic Microbiome Study: Pregnancy Initiative (MOMS-PI) (human)	Liao et al.^56^	NIH SRA PRJNA430482; dbGaP: phs001523
Cervical cerclage using multifilament braided suture induces vaginal dysbiosis, inflammation and poor pregnancy outcome	Kindinger et al.^57^	NIH SRA PRJEB11895
The interaction between the vaginal microbiome, cervical length and vaginal progesterone treatment for preterm birth risk	Kindinger et al.^58^	NIH SRA PRJEB12577
Vaginal microbial dysbiosis increases risk of preterm pre-labor rupture of the fetal membranes, funisitis and neonatal sepsis and is adversely effected by oral administration of erythromycin	Brown et al.^59^	NIH SRA PRJEB21325
Establishment of vaginal microbiota composition in early pregnancy and its association with subsequent preterm prelabour rupture of the fetal membranes	Brown et al.^51^	NIH SRA PRJEB30642
Revealing the role of the cervico-vaginal microbiome in spontaneous preterm birth (human)	Kindinger et al.^58^	NIH SRA: PRJNA504518; dbGAP: phs001739.v1.p1
Tokenized and harmonized Premature Birth Dream Challenge Vaginal Microbiome Data.	This manuscript	March of Dimes Prematurity Database SDY2187

**Software and algorithms**		

MaLiAmPi	Minot et al.^47^	https://zenodo.org/doi/10.5281/zenodo.10015300; https://github.com/jgolob/maliampi

### Resource availability

#### Lead contact

Further information and requests for resources and reagents should be directed to and will be fulfilled by the lead contact, Marina Sirota (marina.sirota@ucsf.edu).

#### Materials availability

• This study did not generate new unique reagents.

#### Data and code availability

• All tokenized and harmonized training and validation data used for this study, including paired metadata and outcomes data is available at pretermdb.org under accession SDY2187.
• Sequence data and associated metadata for Study SDY465 were downloaded from ImmPort^60^ via the March of Dimes Preterm Birth database.^41^ Sequence data and associated metadata for BioProjects SRA: PRJNA242473, SRA: PRJNA294119, SRA: PRJNA393472, and SRA: PRJNA430482 were downloaded from the NCBI Sequence Read Archive.^61^ Additional associated metadata for SRA: PRJNA430482 were requested through and obtained from the RAMS Registry https://ramsregistry.vcu.edu.
• Sequence data and associated metadata for Projects SRA: PRJEB11895, SRA: PRJEB12577, SRA: PRJEB21325, and SRA: PRJEB30642 were downloaded from the Sequence Read Archive of the European Nucleotide Archive,^62^ with associated metadata for SRA: PRJEB11895 and SRA: PRJEB12577 downloaded from Additional Files 4 and 6 from the paper by the Kindinger et al.^58^ Additional associated metadata for Projects SRA: PRJEB11895, SRA: PRJEB12577, SRA: PRJEB21325, and SRA: PRJEB30642 were requested from the senior author.
• Sequence data and associated metadata for accession number phs001739.v1.p1 were downloaded from the database of Genotypes and Phenotypes (dbGaP).^40^.
• The training dataset representing 7 of the 9 aggregated studies and the validation dataset for our Challenge are available under Study ID SDY2187 from the MOD Preterm Birth Research Database (https://pretermbirthdb.org/mod/studydata). Two of the nine training data (SRA: PRJNA430482 and phs001739.v1.p1.) are exclusively available via dbGap after following the application procedures there.
• The processed dataset is also available as a visualization Rshiny application VMAP (Vaginal Microbiome in Pregnancy) – http://vmapapp.org (Figure S11).
• The code for the microbiome data harmonization tool, MaLiAmPi, is available at https://github.com/jgolob/maliampi.
• AM challenge participants’ code for sub-challenge 1 and sub-challenge 2 is in their docker submissions which may be accessed by the hyperlinks listed in Tables S2 and S3, respectively, of this work.
• Any additional information required to reanalyze the data reported in this paper is available from the lead contact upon request.

### Experimental model and study participant details

Collection, generation, and analysis of vaginal microbiome data was approved by the National Heart, Lung, and Blood Institute (NHLBI) Clinical Data Science Institutional Review Board (CDS-IRB) in study number 2021-040, and reliance was granted to the NHLBI CDS-IRB by the University of California, San Francisco Institutional Review Board in study number 21–35274.

#### Validation data generation

##### Wayne State University

###### Study design, sample collection

The microbiome dataset from Wayne State University School of Medicine included in the challenge was a subset of randomly selected 20 cases and 40 controls from a larger retrospective longitudinal case-control study described in detail elsewhere (https://www.researchsquare.com/article/rs-2359402/v1)64. The 20 spontaneous PTB cases included both spontaneous preterm labor with intact membranes (PTL) and preterm prelabor rupture of membranes (PPROM) resulting in delivery 20–36+6 weeks. Cases had 3 or 4 longitudinal samples collected from 10 to 36 weeks of gestation which were matched with samples from controls (2–4 samples per patient). Term controls were defined as women who delivered between 38 and 42 weeks of gestation without congenital anomalies or obstetrical, medical, or surgical complications. Samples of vaginal fluid were collected using a Dacron swab (Medical Packaging Corp., Camarillo, CA). Vaginal swabs were stored at −80°C until time of DNA extraction, following established standard operating procedures. The study was conducted at the Perinatology Research Branch, an intramural program of the Eunice Kennedy Shriver National Institute of Child Health and Human Development, National Institutes of Health, U.S. Department of Health and Human Services, Wayne State University (Detroit, MI), and the Detroit Medical Center (Detroit, MI). The collection of samples was approved by the Institutional Review Boards of the National Institute of Child Health and Human Development and Wayne State University (#110605MP2F(RCR)). All participating women provided written informed consent prior to sample collection.

###### DNA extraction from vaginal swabs

Genomic DNA was extracted from vaginal swabs using a Qiagen MagAttract PowerMicrobiome DNA/RNA EP extraction kit (Qiagen, Germantown, MD), with minor modifications to the manufacturer’s protocols as described in (https://www.researchsquare.com/article/rs-2359402/v1). The purified DNA was transferred to the provided 96-well microplates and stored at −20°C.

###### 16S rRNA gene sequencing and processing

The V4 region of the 16S rRNA gene was amplified from vaginal swab and control DNA extracts and sequenced at Michigan State University’s Research Technology Support Facility (https://rtsf.natsci.msu.edu/) using the dual indexing sequencing strategy developed by Kozich et al.^63^ The forward primer was 515F: 5′-GTGCCAGCMGCCGCGGTAA-3′ and the reverse primer was 806R: 5′-GGACTACHVGGGTWTCTAAT-3’.

##### Stanford university

###### Study design, sample collection

The Stanford University microbiome dataset included in the challenge consisted of 40 cases and 48 controls from a repository of specimens from women enrolled in a longitudinal study conducted by the March of Dimes Prematurity Research Center at Stanford University. Samples of vaginal fluid were collected using a 2x Sterile Catch-All Sample Collection Swab (Epicentre Biotechnologies #QEC091H, Madison, WI). Vaginal swabs were placed into tubes then immediately placed on ice or in a household freezer (−20°C). After samples arrived at the March of Dimes Prematurity Center they were immediately placed on dry ice, inventoried, and then stored at −80 °C at the Stevenson Laboratory until time of DNA extraction. The study was conducted at Stanford Hospital and Clinics. The collection of samples was approved by the Institutional Review Board of Stanford University (Study number 21956). All participating women provided written informed consent prior to sample collection.

###### Vaginal swab DNA extraction and 16S rRNA sequencing

Genomic DNA extraction and microbial sequencing were performed at the Microbial Genomics CoLab Plug-in Facility within the Benioff Center for Microbiome Medicine at University of California, San Francisco. First, vaginal swabs were aseptically transferred to 2 mL tubes pre-filled with 300 μL sterile molecular-grade water. Vaginal samples were vortexed with the swab remaining in the tube. 200 μL vaginal suspension from the tube was withdrawn for downstream processing using the QIAamp BiOstic DNA Kit (QIAGEN, Hilden, Germany). DNA from all samples and several extraction blanks were extracted according to the manufacturer’s protocol and eluted in 50 μL EB buffer. DNA concentrations were quantified using the Qubit dsDNA HS Assay Kit (ThermoFisher Scientific, MA), diluted to 5 ng/μL and stored at −20°C.

The V4 hypervariable region of the 16S rRNA gene was amplified using 515F and 806R primers^64^ with PCR conditions previously described.^65^ Amplicon reactions were quantified using the Qubit dsDNA HS Assay Kit (ThermoFisher Scientific, MA), and pooled at equimolar concentrations. The pooled library was cleaned and concentrated using the Agencourt AMPure XP beads (Beckman-Coulter), quality checked with the Bioanalyzer DNA 1000 Kit (Agilent, Santa Clara, CA), quantified using the KAPA Library Quantification Kit (KAPA Biosystems), and diluted to 2 nM. Library was denatured according to manufacturer’s protocol and spiked in with 40% PhiX control prior to loading onto the NextSeq 550 platform (Illumina, San Diego, CA) for 2 × 150bp sequencing.

### Method details

#### Training data acquisition and processing

The following vaginal microbiome studies were identified by leveraging the March of Dimes Preterm Birth database,^41^ the NCBI Sequence Read Archive,^61^ the European Nucleotide Archive,^62^ and the database of Genotypes and Phenotypes (dbGaP).^40^ Sequence data and associated metadata for the DiGiulio et al.^26^ cohort were downloaded from ImmPort,^60^ under Study SDY465 in May 2016. Sequence data and associated metadata for Romero et al.^54^ cohort were downloaded from the NCBI Sequence Read Archive under BioProject SRA: PRJNA242473 in May 2016. Sequence data and associated metadata for the Callahan et al.^27^ cohort were downloaded from the NCBI Sequence Read Archive under BioProject SRA: PRJNA393472 in January 2018. Sequence data and associated metadata for the Stout et al.^66^ cohort were downloaded from the NCBI Sequence Read Archive under BioProject SRA: PRJNA294119 in January 2018. Sequence data for the Kindinger et al.^58^ cohort were downloaded from the Sequence Read Archive of the European Nucleotide Archive under Projects SRA: PRJEB11895 and SRA: PRJEB12577 in June 2020, and associated metadata was downloaded from Additional Files 4 and 6 from the paper with some additional metadata requested from the senior author. Sequence data and associated metadata for the Brown et al. (2018)^59^ cohort were downloaded from the Sequence Read Archive of the European Nucleotide Archive under Project SRA: PRJEB21325 in June 2020 with some additional metadata requested from the senior author. Sequence data and associated metadata for the Brown et al. (2019)^51^ cohort were downloaded from the Sequence Read Archive of the European Nucleotide Archive under Project SRA: PRJEB30642 in June 2020 with some additional metadata requested from the senior author. Sequence data and associated metadata for the Elovitz et al.^67^ cohort were downloaded from the database of Genotypes and Phenotypes (dbGaP)^40^ under accession number phs001739.v1.p1 in September 2021. Sequence data and associated metadata for the Fettweis et al.^55^ cohort were downloaded from the NCBI Sequence Read Archive under BioProject ID SRA: PRJNA430482 in January 2022, and associated metadata were requested through and obtained from the RAMS Registry.

#### Data processing and harmonization

We applied MaLiAmPi^42^ to both training and test data to process and aggregate the datasets. In brief, MaLiAmPi uses DADA2 to assemble each project’s raw reads into amplicon sequence variants (ASVs). These ASVs are used to recruit full-length 16s rRNA gene alleles from a repository of cached 16S rRNA alleles derived from the NCBI NT database. The objective is to recruit ten full-length 16S rRNA alleles for each ASV with equal sequence identity to the ASV (e.g., bounded best hits), with most ASVs recruiting multiple 16s rRNA alleles with 100% sequence identity for the region of the ASV. These recruits are assembled into a *de novo* maximum-likelihood phylogeny with RAxML and the ASVs are placed onto this common phylogenetic tree with EPA-ng. Finally, these placements are used to determine the alpha-diversity of communities (diversity measures include Shannon, Inverse Simpson, Balance weighted phylogenetic diversity (bwpd), phylogenetic entropy, quadratic, unrooted phylogenetic diversity, and rooted phylogenetic diversity) via the *guppy* utility in the *pplacer* package,^44^ phylogenetic (KR) distance between communities,^68^ provide taxonomic assignments (via the *guppy* ‘hybrid 2’ classifer) to each ASV, and cluster ASVs into phylotypes (based on phylogenetic distance between ASVs). Sequence variance counts were also determined. In addition, VALENCIA^69^ was used to provide the community state type (CST) of each sample and alluvial plots were made using the ggalluvial R package^70^ in order to visualize CST composition by trimester. UMAP representations of the data and violin plots of Shannon alpha diversity before and after processing of the data with MaLiAmPi were visualized to gauge data harmonization. Extensive use of the Python seaborn visualization package was used for figure preparation.

#### DREAM challenge

##### Overall challenge structure

The overview of the Challenge is shown in Figure 1. All Challenge elements were supported by the Synapse platform (http://www.synapse.org), including documentation, access to the data, submission of models, leaderboards, and the discussion forum. To gain access to the data, teams were required to comply with a data use agreement, restricting use of the data outside the Challenge and providing guidelines on ethical participation in the Challenge. Teams were provided the training data, they built their models, dockerized their environment, and submitted their models through the Synapse platform. Models were run on the test data and performance metrics were returned to the teams. Teams were limited to 5 total submissions with the top performing model selected as the final submission to be scored and ranked. Leaderboards were provided throughout the open phase of the Challenge, which provided teams with real-time feedback and comparative performance rankings. After the close of the Challenge, models were evaluated for completeness and reproducibility. For teams to be included in the Preterm Birth DREAM Community, they were required to make the code public, provide a method write-up, and participate in a post-challenge survey to collect information on method development and features of the data important to the model.

##### Participant engagement

Information about our challenge was shared through the Dream Challenges website (https://dreamchallenges.org). Challenge organizers also shared information about the challenge through listservs such as ML-news Google News Group and social media outlets including Facebook, LinkedIn, Reddit, and Twitter.

In order to preserve model environments for portability of models, we required participants to submit Docker environments. These environments contain the necessary programming dependencies and models for each sub-challenge that can run on a processed and prepared microbiome dataset folder arranged in a standardized format. The organizers prepared an example Docker container for participants to utilize as a starting template and held occasional seminars to describe the data and answer questions from participants. Organizers also engaged with participants through the forums to help answer questions throughout the challenge.

#### Sub-challenge 1 - Top performing teams

##### Team UWisc-Madison

For predicting PTB, a LightGBM-based pipeline was built using an ensemble strategy tailored for vaginal microbiome data collected from multiple projects. The model was developed using specimens collected no later than 32 weeks of gestation and included five types of features: counts of taxa at different taxonomic levels, counts of phylotypes, microbiome community states, alpha diversity metrics, and metadata (age, collection week, and race). In particular, the counts of taxa at the family, genus, and species levels, the counts of phylotypes defined at phylogenetic distances of 0.5 and 1, and the alpha diversity metrics including Shannon index, Inverse Simpson Index, phylogenetic entropy, balance-weighted phylogenetic diversity, and rooted/unrooted/quadratic phylogenetic diversity were used. To obtain scale-invariant values, the centered log-ratio (CLR) transformation^71^ was applied to each type of the microbiome count data. Rare microbial features with less than 5 non zero counts in any of the studies of the training set were removed. The LightGBM model was chosen as the prediction model due to its well-known efficiency.^72^ Each specimen was one training sample and each training sample had a total of 1,991 features. 5-fold cross-validation on the subject level was used to tune hyperparameters. Because Project G had a very different sequencing depth profile (the average sequencing depth of Project G is 185,010, whereas the value is below 50,000 for other projects), two prediction models were built: one was trained using specimens from all projects (Model 1) and one was trained only using specimens from Project G (Model 2). When making a prediction given a specimen, the ensembling weights of Model 1 and Model 2 were generated by a logistic regression model with sequencing depth and collection week as features. As one subject is likely to have multiple vaginal microbiome specimens, a customized weighting method was designed to aggregate predictions from multiple specimens on one subject. If a subject has multiple specimens, then the weight of each specimen equals the collection week of the specimen divided by the sum of the collection weeks of all specimens from the subject. In other words, the closer a sample was to delivery, the more impact it would make on the final prediction. The architecture of the pipeline is presented in Figure S8. This pipeline achieved an AUROC of 0.69 and an AUPRC of 0.58 when tested on the validation dataset for sub-challenge 1.

##### Team AI4knowledgeLAB

To predict the risk of PTB, a workflow based on an ensemble of random forest^73^ models with oversampling of the minority class had been used. For the implementation of the model, both metadata and characteristic data of the vaginal microbiome were used. Concerning metadata, information on race and ethnicity and the gestational week when the sample was collected were included into the analysis. Microbiome data included: relative abundances of clusters of variants measured at three different phylogenetic distances (0.1, 0.5, 1), alpha-diversity metrics, and “VALENCIA Community State Types” (CST). The pipeline is shown in Figure S9.

The first step was to eliminate samples collected after the 32 nd week of gestation. A model was then built that takes three different matrices as input, one for each phylogenetic distance, to create three independent models that can output three different predictions for the same individual, which are then combined using an ensemble strategy. Each input matrix had a number of features of 9743, 3651, and 1871: to each matrix of relative abundance of phylotypes were added features related to: alpha-diversity (7), CST (11), and demographics (8).

To make the dataset more balanced, a data augmentation algorithm, SMOTE (Synthetic Minority Over-sampling Technique),^74^ was adopted. As a classification algorithm, random forest was chosen using the default parameters of the Scikit-learn python package^46^ due to its efficiency in handling datasets with a high number of features.^75^ The final output was obtained as the average of the three probability values and the associated class was obtained from the probability value by imposing the classic threshold of 0.5. The prediction model achieved an AUROC of 0.64 and an AUPRC of 0.48 on the Dream Challenge validation dataset.

#### Sub-challenge 2 - Top performing teams

##### Team techtmann lab

To predict early PTB, a basic random forest classifier was employed using python’s Scikit-learn package.^46^ Training data included relative abundances clustered phylogenetically at a distance of 0.1, race of the patient, VALENCIA community state types, diversity metrics, and collection week. This model used default Scikit-learn parameters and involved no additional feature selection or hyperparameter tuning. When tested on the competition validation dataset, the model reported an AUROC of 0.87 and an AUPRC of 0.45.

When investigating feature importance diversity metrics, race, community state type, sample collection week, and some phylotypes were found to be the most important features in the model’s decision-making. Specifically, five phylotypes whose relative abundances were identified as important to predict early PTB: *Lactobacillus jensenii, Lactobacillus iners, Lactobacillus crispatus, Prevotella bivia, and Ureaplasma urealyticum*. This approach is hypothesized to result in a model that was not over-tuned to the training data, allowing it to generalize well to the competition validation dataset.

##### Team KBJ

With the approach of team KBJ for sub-challenge 2, several processes were applied to improve the model prediction performance (Figure S10). First, samples were filtered out by collection week conditions as the test dataset and aggregated all corresponding features. Here, one feature type was selected among several for taxonomy and phylotypes – genus-level and 0.1 phylogenetic distance, respectively. Also, race information was considered, while pairwise distance was excluded. Next, significant features were selected using the minimum redundancy maximum relevance,^76^ which considers mutual information of features in terms of response variables (i.e., early preterm versus non-preterm). The feature selection was conducted for phylotypes, sequence variants, and taxonomy whose dimensions are relatively large compared to the data size. Then, an ensemble model was constructed with five algorithms (Linear Support Vector Classification,^77^ Support Vector Classification,^77^ Quadratic Discriminant Analysis,^78^ Calibrated Classifier,^79^ and Passive Aggressive Classifier^80^) that solely performed the best in cross-validation. All compared models were tested with default parameters by the Lazy Predict^81^ and Scikit-learn^46^ python packages. The prediction model constructed by team KBJ achieved an AUROC of 0.841 and an AUPRC of 0.270 on the Dream Challenge validation dataset. Specifically, the model showed good balanced accuracy (sensitivity: 0.77; specificity: 0.79).

### Quantification and statistical analysis

Performance metrics that were used to evaluate the teams include Area under the receiver operator characteristic (AUROC) curve and Area under the precision-recall (AUPR) curve. On the held-out external validation dataset, metrics of accuracy, sensitivity, and specificity were also computed. These metrics were shown on the final public rankings.

The reproducibility of models, including the baseline, were determined by calculating the Bayes factor for 1000 bootstrapped iterations on a random sampling of the data. For each sub-challenge, the best-performing models from each team were rerun to obtain scores on the random sampling. These scores were then used to calculate the Bayes factor, using the computeBayesFactor function from the challenge scoring R package,^82^ comparing them to the top-performing model as well as the baseline model.

To increase our certainty of DREAM Challenge participants’ rankings whose models’ performances could have been affected by prediction threshold and class imbalance in our validation dataset, we employed the following strategies to validate participants’ models for both sub-challenges on the external dataset: inverting labels, bootstrapped random subsampling, bootstrapped under-sampling, and bootstrapped over-sampling.

#### Inverted labels

Invert the class labels for the external dataset and prediction model outputs (i.e., classifying preterm or early preterm births as term births, and vice versa), and computing AUROC/AUPR curves.

#### Bootstrapped random subsampling

Randomly sample a subset of 100 from the 152 participants of the external dataset, and run the prediction models on the validation data subset, bootstrapped 1000 times.

#### Bootstrapped undersampling

Undersample the external dataset (n = 152) to balance the minority (Preterm, n = 63. Early preterm, n = 13) and majority (i.e., Term, n = 89) classes by randomly sampling from the minority and the majority groups to have the same number in each group (n = 50 for Preterm and n = 50 for Term in sub-challenge 1, and n = 13 for Early Preterm and n = 13 for Term in for sub-challenge 2), and then computing AUROC/AUPRC on the undersampled external validation dataset, bootstrapped 1000 times.

#### Bootstrapped oversampling

Oversample the external dataset to balance the preterm or early preterm and term classes by randomly sampling per group (n = 200 for Preterm and n = 200 for Term in sub-challenge 1, and n = 200 for Early Preterm and n = 200 for Term in for sub-challenge 2), and then computing AUROC/AUPRC oversampled external dataset, bootstrapped 1000 times.

Individual team methods are linked to in Table S1.

DREAM challenge participants and teams were surveyed to gather information on how they developed their models.

Sensitivity analysis was carried out removing gestational age at sampling as a feature.

As with previous DREAM Challenges, ensemble models were generated to explore the "wisdom of the crowds" phenomenon, by aggregating the best-performing models from each team. For each sub-challenge, we experimented with 3 ensemble models by calculating the mean estimation from: 1) top two performing models; 2) models with Bayes factor less than 20; 3) all models.

#### Feature permutation

We employed feature permutation to empirically determine which of the microbiome feature sets, and in turn which specific features, models made use of to make their predictions. Feature importance was determined across the best performing models for sub-challenges 1 and 2 that demonstrated predictive performance at threshold of 0.64 for sub-challenge 1 and a threshold of 0.80 sub-challenge 2 which also could be run in a bootstrapped manner in a tractable amount of time (e.g., offer a prediction in under 10 s on a 12 core AMD Ryzen 3900X processor). Three models for sub-challenge 1 and eight models for sub-challenge 2 fit these criteria and were evaluated. We employed a staged approach, first randomizing feature tables to identify which feature tables a model used, and then in those feature table-model pairing, randomized individual features.

#### Table permutation

The alpha-diversity, taxonomy (species-, genus-, and family level), phylotype (1, 0.5, and 0.1 binning distance), VALENCIA CSTs, and raw sequence variant count tables (with features in columns and specimens in rows) were each individually shuffled by row without replacement. After obtaining a baseline prediction from each model with unmodified feature tables, the model was rerun with a shuffled table replacing one of the feature tables and the predictions recorded and compared to the baseline prediction. A feature table was scored as used by that model if the predict changed compared to the baseline prediction.

#### Feature permutation

The results of the table permutation effort as above were then used to filter down to model – feature table pairs. Again a baseline prediction was made, and then each column (feature) was shuffled one-by-one and the model output recorded and compared to the baseline. If the predictions varied when that specific feature was shuffled, it was considered ‘important’ for that model to make its prediction. To reduce the computational load, only features with a density over 10% (e.g., found in at least 10% of the specimens) were considered.

## Supplemental information

Document S1. Figures S1‒S11 and Tables S1‒S5 and S8

Table S6. Feature permutation and univariate correlation analysis of features vs. preterm birth

Table S7. Feature permutation and univariate correlation analysis of features vs. early preterm birth

Document S2. Article plus supplemental information
