# Supplementary material for: Microbiome preterm birth DREAM challenge: Crowdsourcing machine learning approaches to advance preterm birth research
Source: Cell Rep Med. 2023 Dec 21;5(1):101350. doi: 10.1016/j.xcrm.2023.101350 (PMC10829755; doi:10.1016/j.xcrm.2023.101350)
Supplement: Document S1. Figures S1‒S11 and Tables S1‒S5 and S8 [file mmc1.pdf]

Cell Reports Medicine, Volume *5*

Supplemental information

Microbiome preterm birth DREAM challenge: Crowdsourcing machine learning approaches to advance preterm birth research

Jonathan L. Golob, Tomiko T. Oskotsky, Alice S. Tang, Alennie Roldan, Verena Chung, Connie W.Y. Ha, Ronald J. Wong, Kaitlin J. Flynn, Antonio Parraga-Leo, Camilla Wibrand, Samuel S. Minot, Boris Oskotsky, Gaia Andreoletti, Idit Kosti, Julie Bletz, Amber Nelson, Jifan Gao, Zhoujingpeng Wei, Guanhua Chen, Zheng-Zheng Tang, Pierfrancesco Novielli, Donato Romano, Ester Pantaleo, Nicola Amoroso, Alfonso Monaco, Mirco Vacca, Maria De Angelis, Roberto Bellotti, Sabina Tangaro, Abigail Kuntzleman, Isaac Bigcraft, Stephen Techtmann, Daehun Bae, Eunyoung Kim, Jongbum Jeon, Soobok Joe, The Preterm Birth DREAM Community, Kevin R. Theis, Sherrianne Ng, Yun S. Lee, Patricia Diaz-Gimeno, Phillip R. Bennett, David A. MacIntyre, Gustavo Stolovitzky, Susan V. Lynch, Jake Albrecht, Nardhy Gomez-Lopez, Roberto Romero, David K. Stevenson, Nima Aghaeepour, Adi L. Tarca, James C. Costello, and Marina Sirota

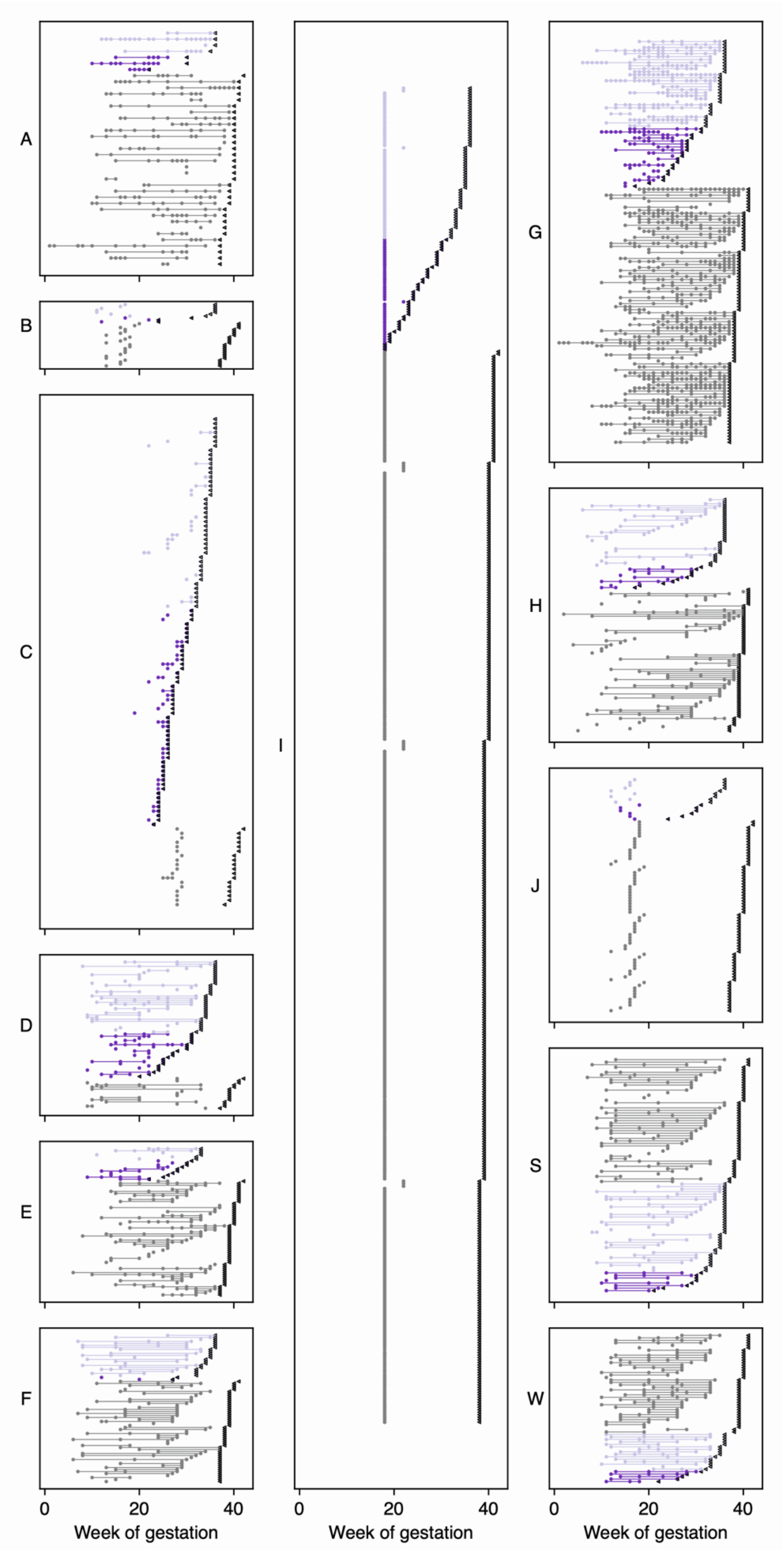

Supplementary Figure 1: Individual study designs. Gestational week at sample collection stratified by study and colored by birth outcome. Related to STAR Methods data availability and Figure 1.

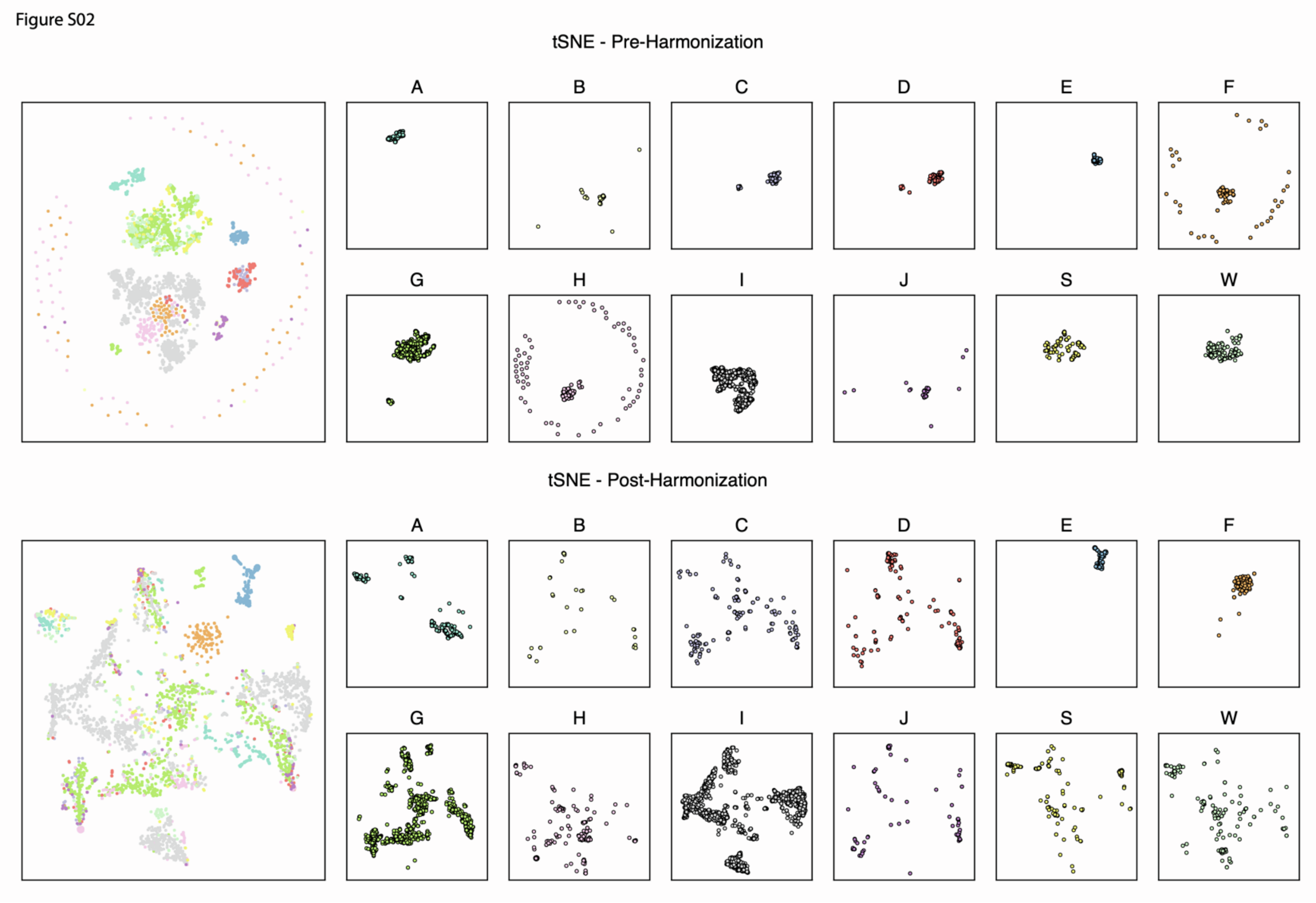

Supplementary Figure 2: tSNE ordination plots based on Bray-Curtis distance. Pseudo counts were generated (normalized to 10,000 reads per specimen) based on either sequence variant or phylotype counts and used to calculate pairwise distance between specimens. This pairwise distance matrix was used for tSNE ordination, with a default perlexity of 30. Related to Figure 2.

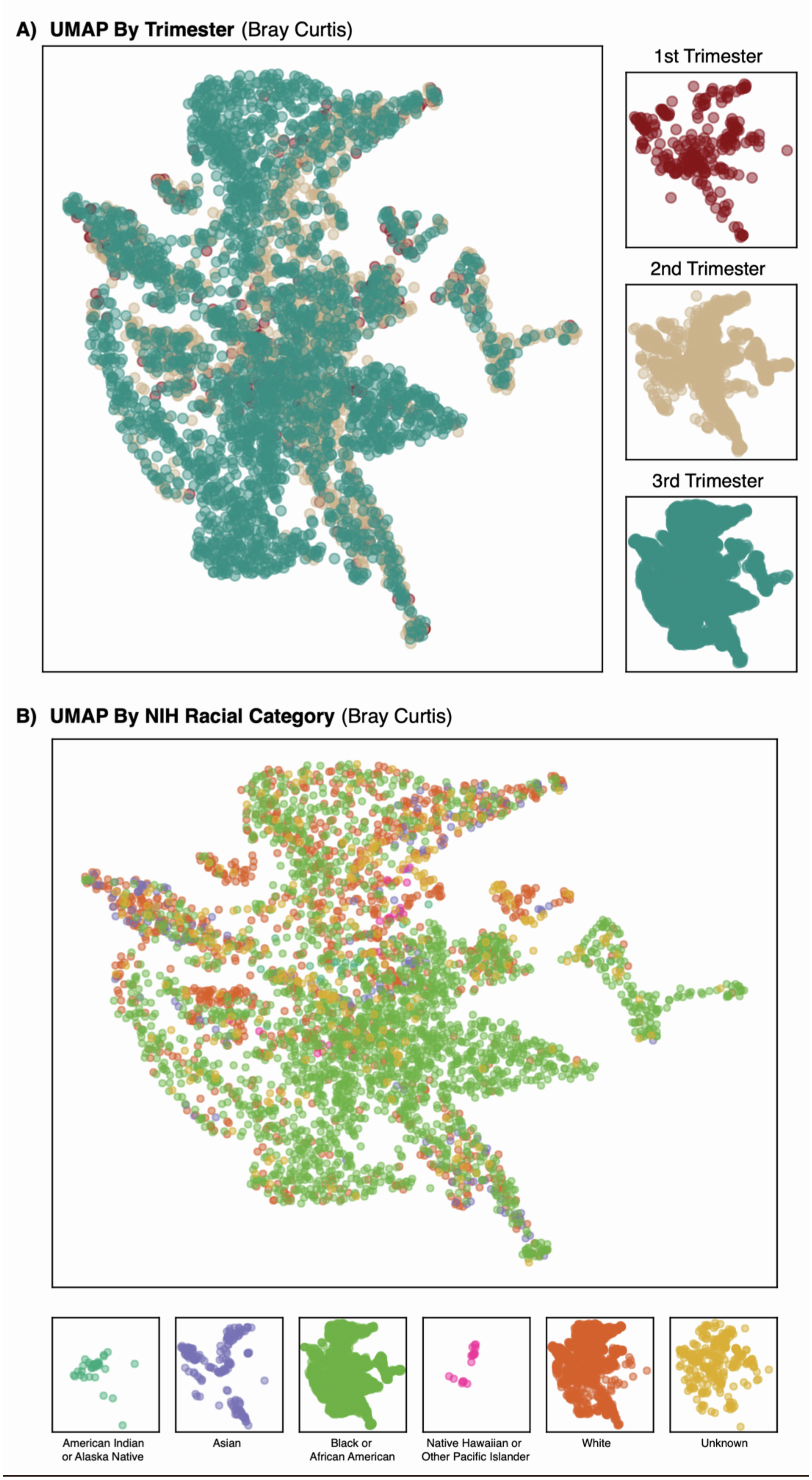

Supplementary Figure 3: UMAP ordination plots of data after harmonization, where each dot represents one vaginal microbiome sample, colored a) by trimester and b) by race/ethnicity. Related to figure 2.

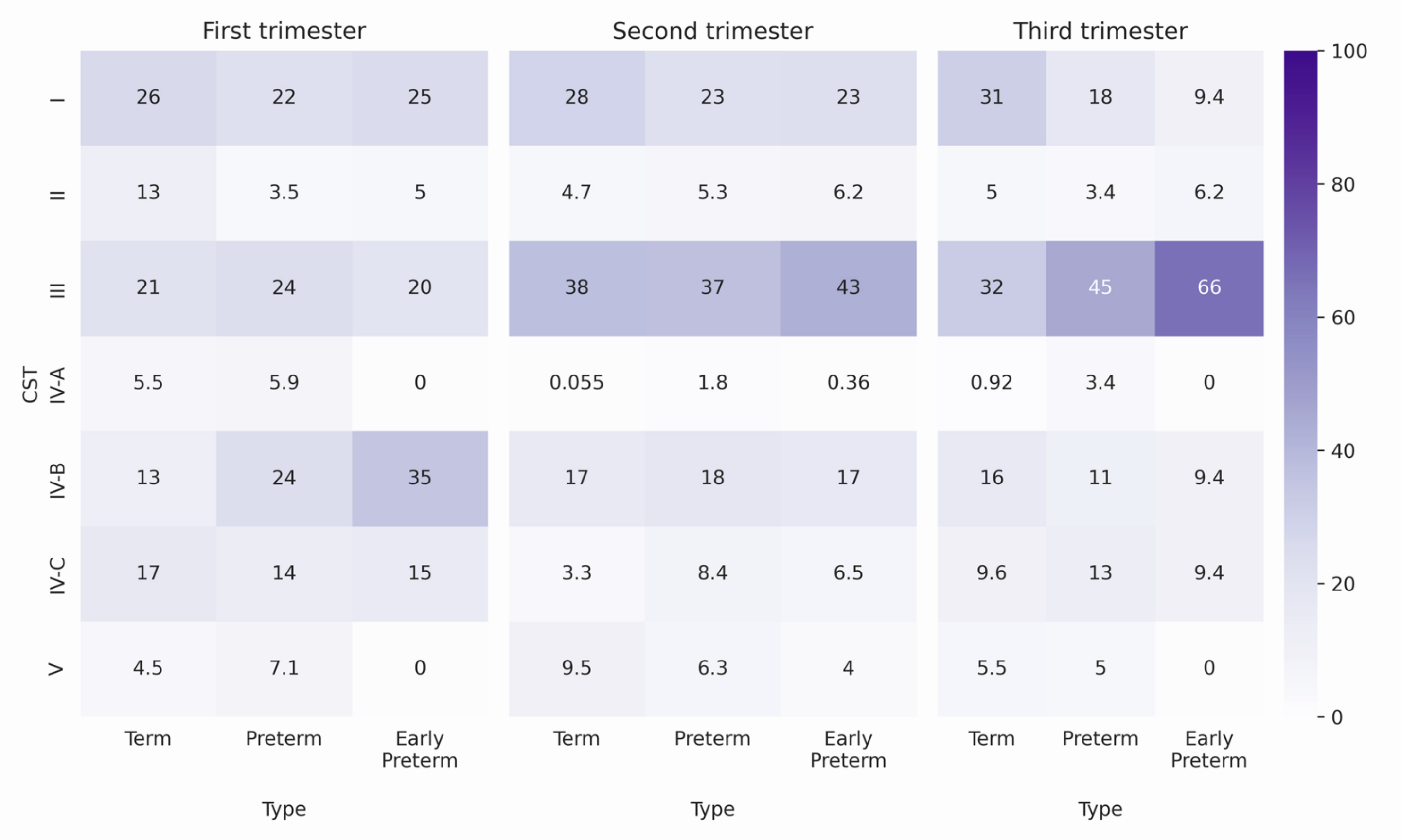

Supplementary Figure 4: CST heatmap. Heatmap of community state types (CST) for term, preterm, and early preterm births across the first, second, and third trimesters of pregnancy. Related to Figure 2.

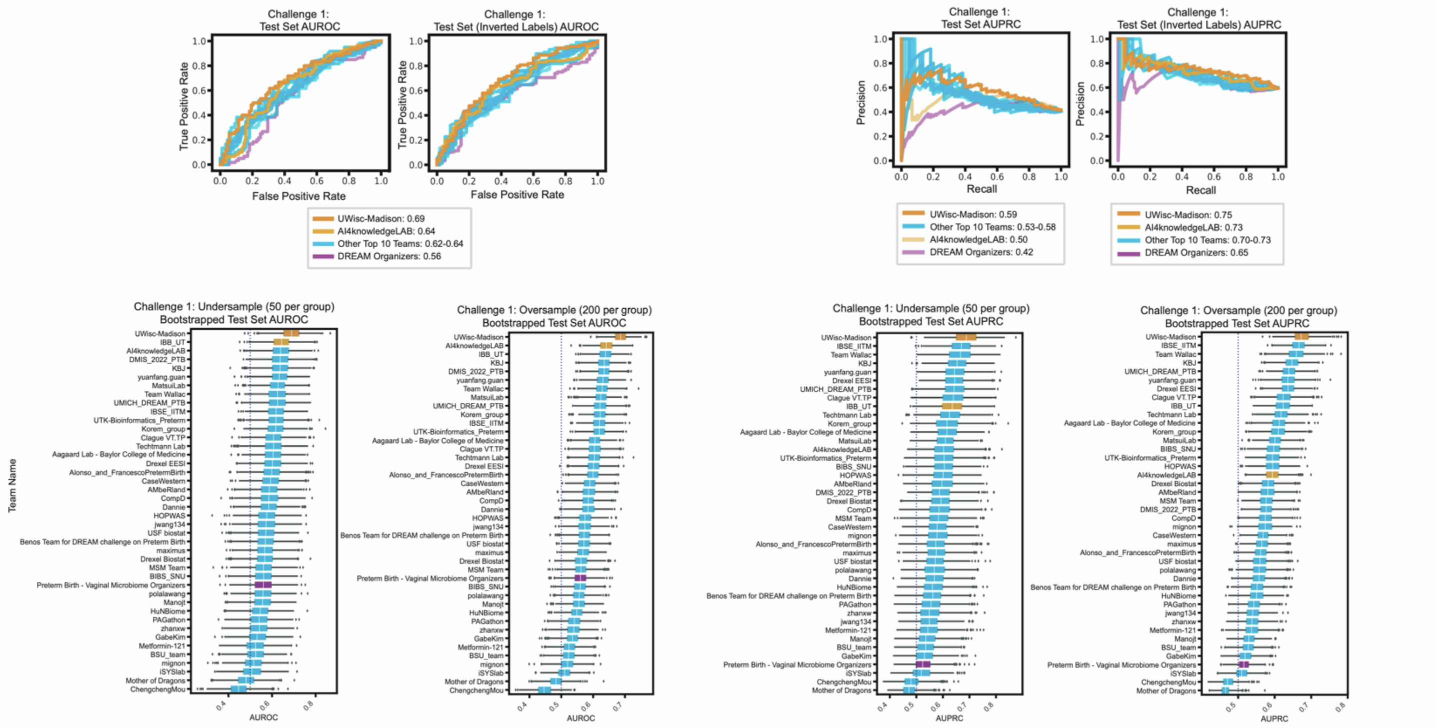

Supplementary Figure 5: Bootstrapped results for sub-challenge 1: preterm birth prediction. Top includes curves for inverted labels in the test set, while bottom includes undersampling and oversampling per group (preterm/term) to ensure balance between groups. Left is AUROC, right is AUPRC. Related to Figure 3.

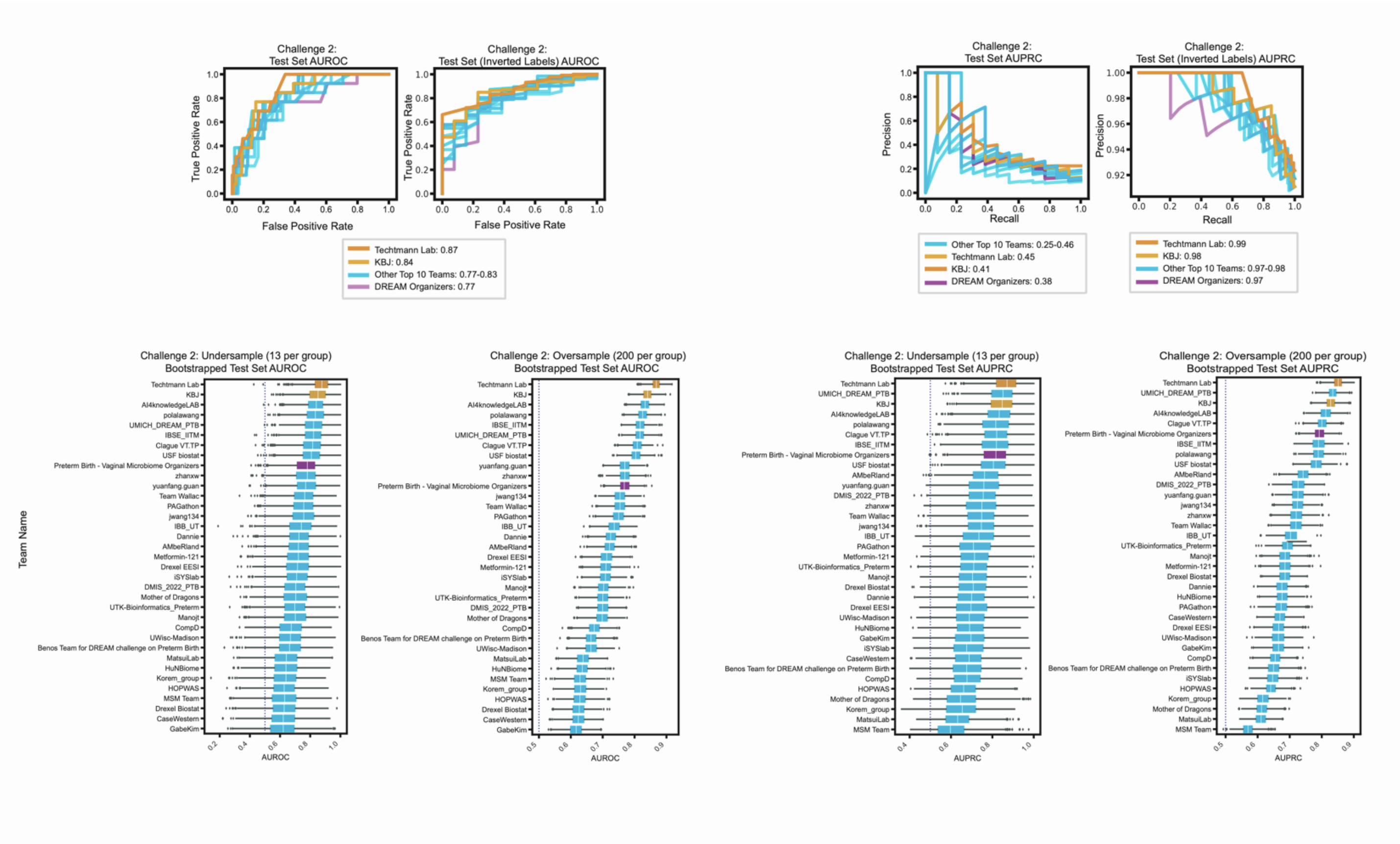

Supplementary Figure 6: Bootstrapped results sub-challenge 2: early preterm birth prediction. Top includes curves for inverted labels in test set, while bottom includes undersampling and oversampling per group (early preterm/not early preterm) to ensure balance between groups. Left is AUROC, right is AUPRC. Related to Figure 3.

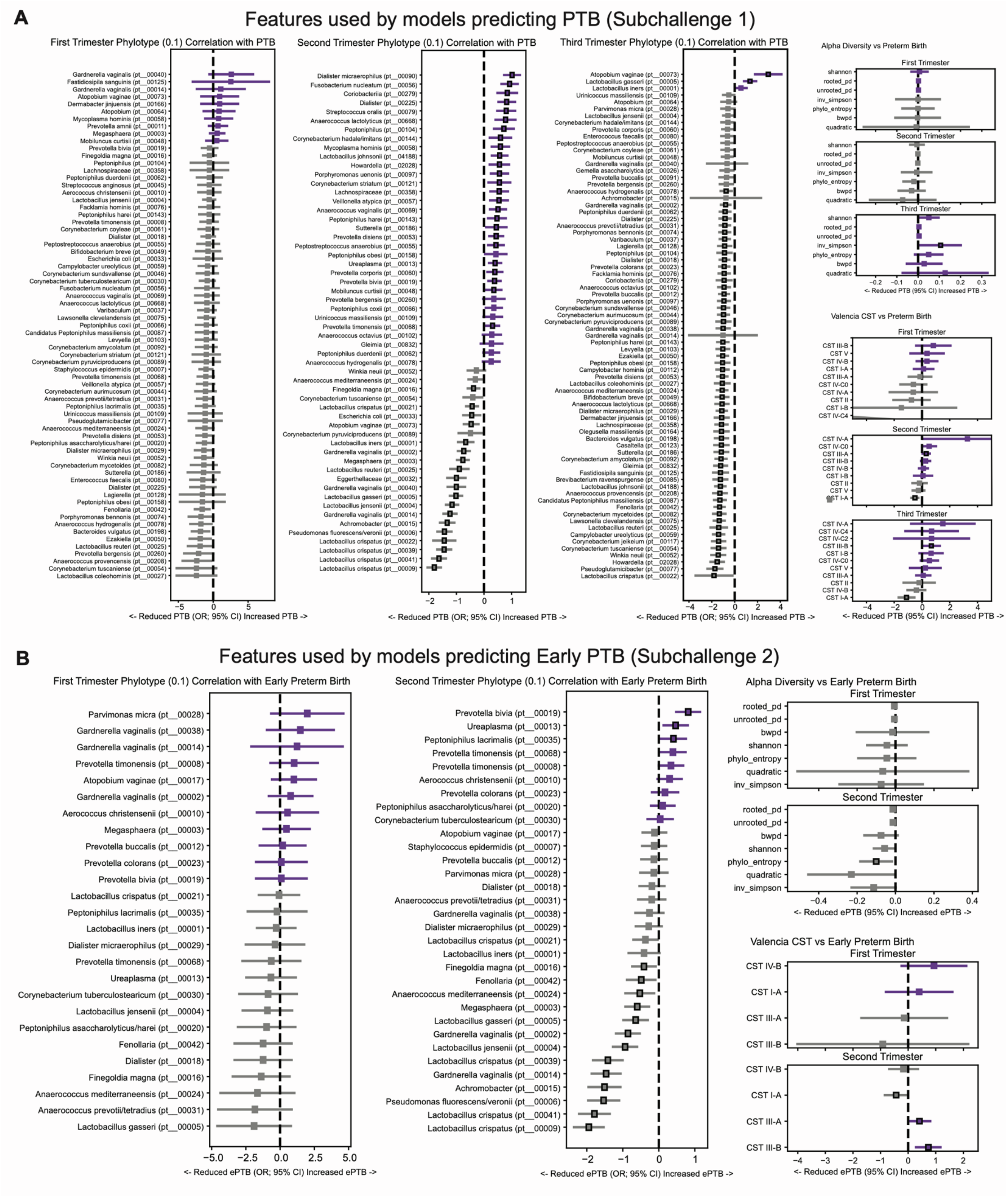

Supplemental Figure 7: Features Across Best Performing Models. For models performing at threshold or above baseline, odds ratios (OR) with 95% confidence intervals (CI) reflecting correlation with PTB by trimester of specific phylotypes (0.1), diversity metrics, and community state types (CSTs) of features used extensively by top-performing models for a) sub-challenge 1 and b) sub-challenge 2. Related to Figure 4.

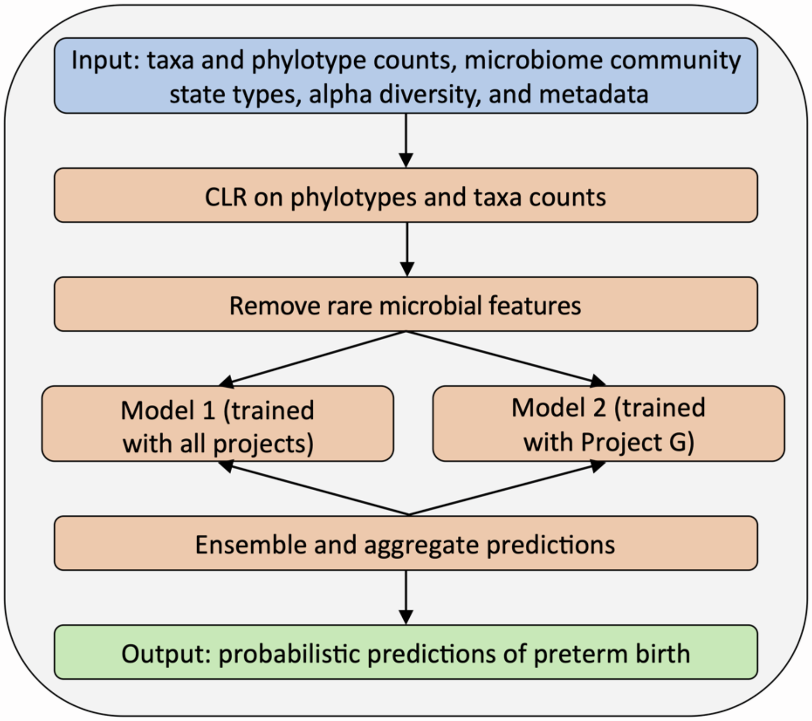

Supplementary Figure 8: Overview of the pipeline of U-Wisconsin team. The architecture of the pipeline for prediction of preterm birth using microbiome data and metadata. CLR is applied to each type of the microbiome count data. Rare microbial features are filtered out. Two LightGBM models are trained: one on all available specimen data (Model 1), and another on data from Project G only (Model 2). The predictions from these models are then combined, and the aggregate prediction is used to generate a probabilistic prediction of preterm birth. Related to STAR Methods.

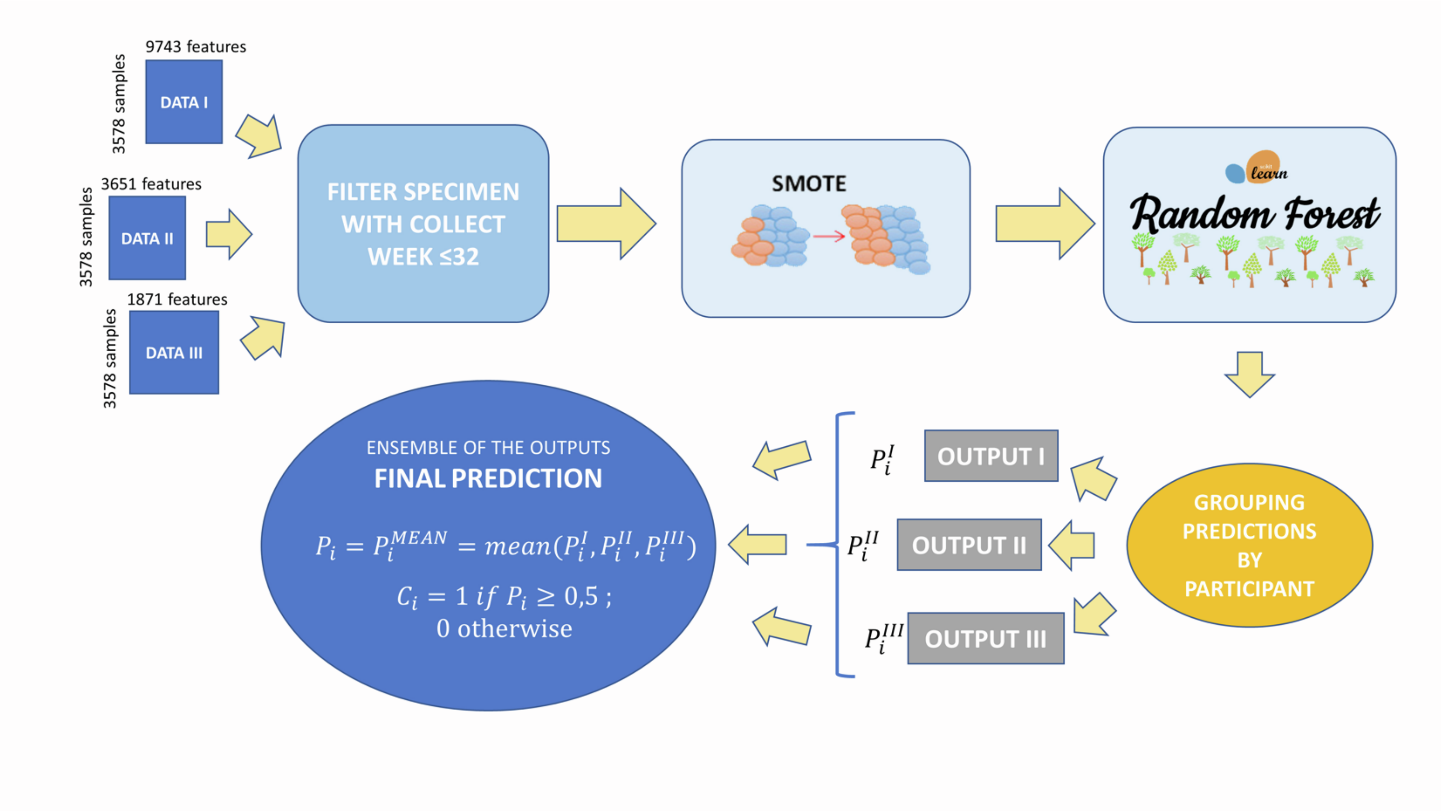

Supplementary Figure 9: Workflow of the analysis by Team AI4knowledgeLAB. The probability score of the final output was obtained as the average of the 3 probability values and the associated class was obtained from the probability value by imposing the classic threshold of 0.5. Related to STAR Methods.

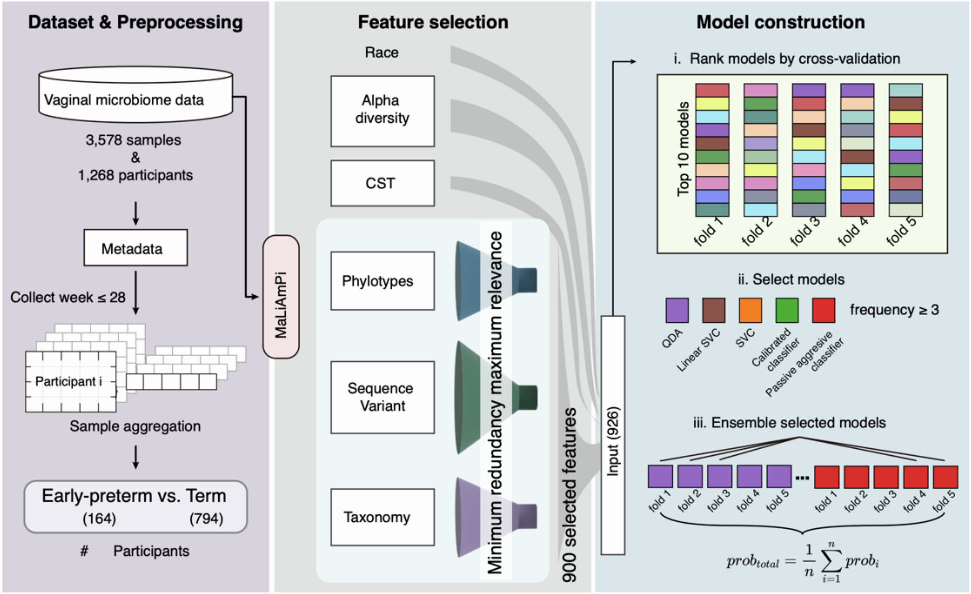

Supplementary Figure 10: Overview of the model of Team KBJ. Left represents preprocessing of provided metadata and processed outputs from MaliAmPi pipeline. They extracted samples according to the test set condition and aggregated features to represent participants. Then, sparse feature types were handled with mRMR and concatenated with other features. Additional race information was used as a feature. For ensemble learning, based on 26 different machine learning models, five algorithms were selected by top-ranked models. The final predicted value was calculated as the mean of each probability. Related to STAR Methods.

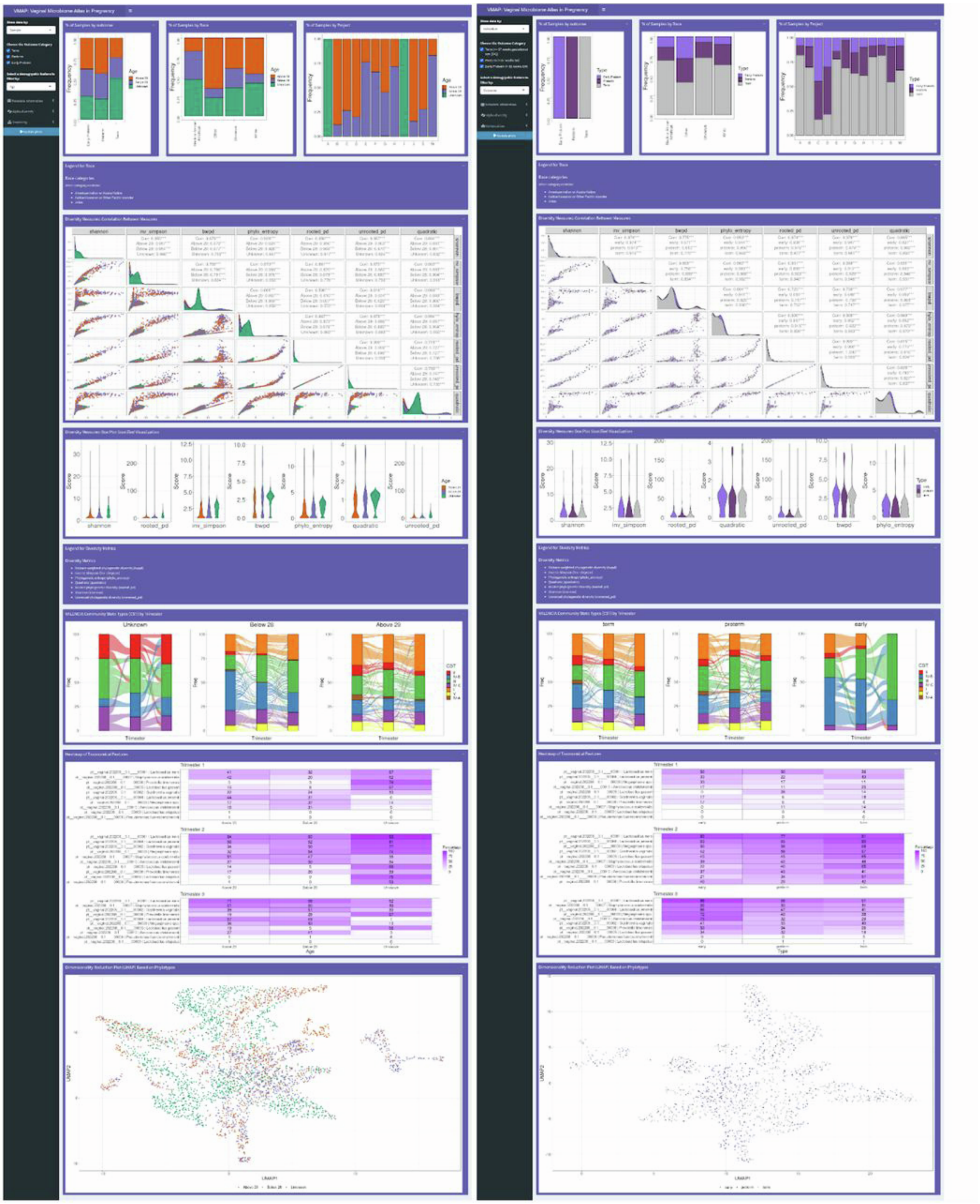

Supplementary Figure 11: VMAP RShiny App Screenshot. A user can visualize demographic features of the cohort, diversity measures (both correlation between them) and each one by outcome of interest, CSTs as alluvial plots, dimensionality reduction plots of phylogeny and heatmaps of taxonomical features. The visualizations are customizable by the bar on the left allowing one to choose which projects and samples to visualize. A) visualized by age B) visualized by outcome. Related to Figure 2 and STAR Methods.

SUPPLEMENTARY TABLES

Supplementary Table 1: Details of the training and validation studies. Summary of participants, samples, and V region sequences of training (A-J) and validation (W and S) datasets. Related to Table 1 and STAR Methods.

Study ID	Study Accession ID	Center	Title (Authors, year)	# of Participants	# of Term | PTB | Early PTB Participants	# of Samples	# of Term|PTB |Early PTB Samples	V Region Sequences	Instrument
A	SDY465	Stanford University	Temporal and spatial variation of the human microbiota during pregnancy (DiGiulio et al., 2015)	39	32|7|3	231	180|51|21	V3 - V5	454 GS FLX Titanium
B	PRJEB11895	Imperial College London	The interaction between vaginal microbiota, cervical length, and vaginal progesterone treatment for preterm birth risk (Kindinger et al., 2017)	33	23|10|3	33	23|10|3	V1 - V3	Illumina MiSeq
J	PRJEB12577	Imperial College London	The interaction between vaginal microbiota, cervical length, and vaginal progesterone treatment for preterm birth risk (Kindinger et al., 2017)	83	68|15|6	83	68|15|6	V1 - V3	Illumina MiSeq
C	PRJEB21325	Imperial College London	Vaginal dysbiosis increases risk of preterm fetal membrane rupture, neonatal sepsis and is exacerbated by erythromycin (Brown et al., 2018)	110	18|92|49	144	20|124|67	V1 - V2	Illumina MiSeq
D	PRJEB30642	Imperial College London	Establishment of vaginal microbiota composition in early pregnancy and its association with subsequent preterm prelabor rupture of the fetal membranes (Brown et al., 2019)	70	15|55|21	134	26|108|38	V1 - V2	Illumina MiSeq
E	PRJNA242473	University of Maryland	The vaginal microbiota of pregnant women who subsequently have spontaneous preterm labor and delivery and those with a normal delivery at term (Romero et al., 2014)	73	57|16|10	168	137|31|19	V1 - V3	454 GS FLX Titanium
F	PRJNA294119	Washington University	Early pregnancy vaginal microbiome trends and preterm birth (Stout et al., 2017)	74	51|23|2	145	99|46|2	V1 - V3	454 GS FLX Titanium
G	PRJNA393472	Stanford University	Replication and Refinement of a Vaginal Microbial Signature of Preterm Birth (Callahan et al, 2017)	134	85|49|20	957	670|287|71	V4	llumina HiSeq 2500
H	PRJNA430482	Virginia Commonwealth	The vaginal microbiome and preterm birth (Fettweis et al.,	114	70|44|11	216	137|79|19	V1–V3	Illumina HiSeq 4000
I	PRJNA504518 (phs001739.v1.p1.)	University of Pennsylvania	Cervicovaginal microbiota and local immune response modulate the risk of spontaneous preterm delivery (Elovitz et al., 2019)	538	432|106|45	1467	1229|238|82	V3 - V4	Illumina HiSeq 2500

S	Not applicable	Stanford University	Not applicable	86	47|39|8	170	94|76|18	V4	Illumina NextSeq 550
W	Not applicable	Wayne State University	*Working title: The Vaginal Microbiota in Early Pregnancy Identifies a Subset of Women at Risk for Early Preterm Prelabor Rupture of Membranes and Preterm Birth*	60	40|20|5	159	102|57|17	V4	Illumina MiSeq
Total	Not applicable	Not applicable	Not applicable	1414	938|476|183	3907	2785|1122|363	V1, V2, V3, V4, +/or V5	454 GS FLX Titanium, Illumina MiSeq, Illumina HiSeq 2500, Illumina HiSeq 4000, or Illumina NextSeq 550

Supplementary Table 2: Complete scores for sub-challenge 1 predicting preterm birth. Related to Figure 3.

Final rank	Team	Docker Submission	Write-up	AUROC	AUPRC	Accuracy	Sensitivity	Specificity	MCC
1	UWisc-Madison	syn34232353	syn34232349	0.688	0.575	0.669	0.483	0.796	0.294
2	AI4knowledgeLAB		syn35802768	0.641	0.484	0.622	0.600	0.636	0.233
3	IBB_UT	syn36382380	syn34085067	0.64	0.526	0.622	0.133	0.955	0.158
4	KBJ	syn36385198	syn35594394	0.635	0.538	0.601	0.483	0.682	0.167
5	DMIS_2022_PTB	syn36448001	syn36245853	0.634	0.47	0.595	0.267	0.818	0.101
6	@yuanfang.guan	syn36463027	syn34555655	0.631	0.52	0.405	1.000	0.000	–
7	Team Wallac	syn35594525	syn35594407	0.629	0.563	0.541	0.667	0.455	0.121
8	MatsuiLab	syn36446946	syn36175341	0.625	0.515	0.642	0.367	0.83	0.223
9	UMICH_DREAM_PTB	syn36470935	syn33585048	0.623	0.540	0.595	0.000	1.000	–
10	Korem_group	syn36366426	syn35461338	0.620	0.498	0.588	0.000	0.989	-0.068
11	UTK-Bioinformatics_Preterm	syn36157749	syn36157104	0.620	0.487	0.601	0.650	0.568	0.214
12	IBSE_IITM	syn36507895	syn36397234	0.619	0.580	0.622	0.300	0.841	0.168

13	Clague VT.TP		syn36495788	0.605	0.523	0.676	0.300	0.932	0.309
14	Aagaard Lab - Baylor College of Medicine - Texas Childrens Hospital	syn35558450	syn35558406	0.604	0.514	0.608	0.183	0.898	0.116
15	Techtmann Lab	syn36503534	syn35558406	0.604	0.530	0.615	0.133	0.943	0.133
16	Drexel EESI	syn36448073	syn36447322	0.602	0.547	0.561	0.667	0.489	0.154
17	Alonso_and_FrancescoPretermBirth	syn35440821	syn35437513	0.599	0.452	NaN	–	–	–
18	CaseWestern	syn36154854	Preterm Birth Prediction-Vaginal Microbiome by CaseWestern	0.590	0.481	0.527	0.800	0.341	0.154
19	AMbeRland	syn36350105	Preterm Birth Prediction-Vaginal Microbiome AMbeRland	0.587	0.472	0.534	0.867	0.307	0.200
20	CompD	syn36472365	Preterm Birth Prediction-Vaginal Microbiome CompD	0.586	0.467	0.601	0.067	0.966	0.075
21	@Dannie		–	0.585	0.436	0.574	0.500	0.625	0.124
22	HOPWAS	syn36486934	Preterm Birth Prediction-Vaginal Microbiome-HOPWAS	0.574	0.496	0.487	0.950	0.171	0.181
23	@jwang134	syn35250016	Preterm Birth Prediction-Vaginal Microbiome jwang134	0.573	0.436	0.534	0.150	0.796	-0.069
24	USF biostat	syn36381878	Preterm Birth Prediction - Microbiome DREAM Challenge USF Biostat	0.572	0.452	0.588	0.000	0.989	-0.068
25	Benos Team for DREAM challenge on Preterm Birth	syn36488106	Preterm Birth Prediction-Vaginal Microbiome (Benos Team for DREAM challenge on Preterm Birth)	0.571	0.438	0.581	0.200	0.841	0.053
26	@maximus		–	0.569	0.462	0.601	0.333	0.784	0.131
27	Drexel Biostat	syn36444097	Preterm Birth Prediction-Vaginal Microbiome-	0.563	0.485	0.588	0.250	0.818	0.082

			Drexel Biostat						
28	MSM Team	syn33995110	Preterm Birth Prediction-Vaginal Microbiome MSM Team	0.561	0.476	0.473	0.700	0.318	0.019
29	Preterm Birth - Vaginal Microbiome Organizers		–	0.561	0.410	0.574	0.000	0.966	-0.119
30	BIBS_SNU		–	0.559	0.503	0.541	0.767	0.386	0.161
31	@polalawang	syn35545064	Preterm Birth Prediction-Vaginal Microbiome polalawang	0.557	0.446	0.581	0.150	0.875	0.036
32	@Manojt	syn36430971	Preterm Birth Prediction-Vaginal Microbiome - Darwin's Finches	0.556	0.418	0.568	0.167	0.841	0.010
33	HuNBiome		–	0.549	0.429	0.541	0.350	0.671	0.021
34	PAGathon	syn36245846	Preterm Birth Prediction-Vaginal Microbiome PAGathon	0.540	0.435	0.595	0.067	0.955	0.046
35	@zhanxw	syn34579624	–	0.540	0.431	0.588	0.083	0.932	0.028
36	@GabeKim		–	0.534	0.408	0.547	0.150	0.818	-0.042
37	Metformin-121	syn35739726	Preterm Birth Prediction-Vaginal Microbiome Metformin-121	0.525	0.427	0.595	0.000	1.000	–
38	BSU_team	syn36467147	Preterm Birth Prediction-Vaginal Microbiome BSU_team	0.525	0.430	0.547	0.083	0.864	-0.082
39	@mignon		–	0.518	0.472	0.615	0.167	0.921	0.134
40	iSYSlab	syn36266871	Preterm Birth Prediction-Vaginal Microbiome iSYSLab	0.509	0.399	0.412	0.883	0.091	-0.042
41	Mother of Dragons		–	0.483	0.365	NaN	–	–	–
42	@ChengchengMou		–	0.447	0.373	0.514	0.367	0.614	-0.020

Supplementary Table 3: Complete scores for sub-challenge 2 predicting early preterm birth. Related to Figure 3.

Final rank	Team	Docker Submission	Write-up	AUROC	AUPRC	Accuracy	Sensitivity	Specificity	MCC
1	Techtmann Lab	syn36511607	Preterm Birth Prediction- Vaginal Microbiome Techtmann Lab	0.868	0.446	0.911	0.000	1.000	–
2	KBJ	syn36314881	Preterm Birth Prediction - Microbiome DREAM Challenge Team-KBJ	0.841	0.270	0.788	0.769	0.790	0.363
3	AI4knowledgeLAB	syn36448011	Preterm Birth Prediction - Vaginal Microbiome AI4knowlwdgeLAB	0.831	0.343	0.904	0.231	0.970	0.268
4	@polalawang	syn35545064	Preterm Birth Prediction- Vaginal Microbiome polalawang	0.827	0.297	0.911	0.000	1.000	–
5	IBSE_IITM	syn36472647	Preterm Birth Prediction- Vaginal Microbiome - IBSE_IITM	0.817	0.273	0.904	0.308	0.962	0.320
6	UMICH_DREAM_PTB	syn36336742	Preterm Birth Prediction- Vaginal Microbiome UMICH_DREAM_PTB	0.816	0.442	0.911	0.000	1.000	–
7	Clague VT.TP	syn36502370	Preterm Birth Prediction- Vaginal Microbiome Clague VT.TP - Random Forest V5 - SC2	0.807	0.350	0.904	0.231	0.970	0.268
8	USF biostat	syn36472987	Preterm Birth Prediction - Microbiome DREAM Challenge USF Biostat	0.803	0.272	0.911	0.000	1.000	–
9	@yuanfang.guan	syn35365964	preterm_guan	0.769	0.189	0.192	1.000	0.113	0.106
10	@zhanxw	syn34579632	–	0.768	0.173	0.904	0.000	0.993	-0.026
11	Preterm Birth - Vaginal Microbiome Organizers		–	0.768	0.373	0.911	0.000	1.000	–
12	Team Wallac	syn35657505	Preterm Birth Prediction- Vaginal Microbiome Team Wallac	0.754	0.185	0.719	0.615	0.729	0.214
13	@jwang134	syn35250016	Preterm Birth Prediction- Vaginal Microbiome jwang134	0.753	0.203	0.911	0.000	1.000	–

14	PAGathon	syn36245846	Preterm Birth Prediction- Vaginal Microbiome PAGathon	0.751	0.158	0.911	0.000	1.000	–
15	IBB_UT	syn36444486	Preterm Birth Prediction- Vaginal Microbiome IBB_UT	0.736	0.176	0.911	0.000	1.000	–
16	@Dannie		–	0.725	0.149	0.904	0.000	0.993	-0.026
17	AMbeRland	syn36352211	Preterm Birth Prediction- Vaginal Microbiome AMbeRland	0.720	0.215	0.740	0.462	0.767	0.150
18	Drexel EESI	syn36448030	Preterm Birth Prediction- Vaginal Microbiome Drexel EESI	0.711	0.146	0.288	0.923	0.226	0.104
19	Metformin-121	syn36118224	Preterm Birth Prediction- Vaginal Microbiome Metformin-121	0.710	0.216	0.911	0.000	1.000	–
20	iSYSlab	syn36266871	Preterm Birth Prediction- Vaginal Microbiome iSYSLab	0.709	0.139	0.843	0.154	0.910	0.062
21	@Manojt	syn36445483	Preterm Birth Prediction- Vaginal Microbiome - Darwin's Finches	0.703	0.157	0.897	0.000	0.985	-0.037
22	DMIS_2022_PTB	syn36448401	Preterm Birth Prediction- Vaginal Microbiome DMIS_2022_PTB	0.701	0.207	0.699	0.615	0.707	0.196
23	UTK-Bioinformatics_Preterm		Preterm_Challenge_UTK	0.700	0.224	0.911	0.000	1.000	–
24	Mother of Dragons		–	0.697	0.125	NaN	–	–	–
25	CompD	syn36472371	Preterm Birth Prediction- Vaginal Microbiome CompD	0.674	0.196	0.911	0.000	1.000	–
26	UWisc-Madison		Preterm Birth Prediction - Vaginal Microbiome - UWisc-Madison - Task 1 - Submission 1	0.664	0.153	0.911	0.000	1.000	–
27	Benos Team for DREAM challenge on Preterm Birth	syn36506184	Preterm Birth Prediction- Vaginal Microbiome (Benos Team for DREAM challenge on Preterm Birth)	0.663	0.136	0.911	0.000	1.000	–
28	MatsuiLab	syn36446946	Preterm Birth Prediction- Vaginal Microbiome MatsuiLab	0.638	0.167	0.322	0.923	0.263	0.123

29	HuNBiome		–	0.635	0.130	0.911	0.000	1.000	–
30	Korem_group	syn36017582	Preterm Birth Prediction- Vaginal Microbiome CUMC	0.630	0.122	0.911	0.000	1.000	–
31	MSM Team	syn35432106	Preterm Birth Prediction- Vaginal Microbiome MSM Team	0.628	0.109	0.637	0.615	0.639	0.149
32	HOPWAS	syn36486934	Preterm Birth Prediction- Vaginal Microbiome-HOPWAS	0.628	0.203	0.096	1.000	0.008	0.026
33	Drexel Biostat	syn36505751	Preterm Birth Prediction- Vaginal Microbiome-Drexel Biostat	0.625	0.172	0.911	0.077	0.993	0.170
34	CaseWestern	syn36245137	Preterm Birth Prediction- Vaginal Microbiome by CaseWestern	0.622	0.255	0.740	0.385	0.774	0.106
35	@GabeKim		–	0.617	0.154	0.897	0.000	0.985	-0.037

Supplementary Table 4: Summary of the approach and methods for the top-performing models for Sub-challenge 1 (preterm birth). Related to STAR Methods and figure 3.

		Performance Metrics	Performance Metrics	
**Team**	**Modeling Methods**	**AUROC**	**AUPRC**	**Hyperparameters**
UWisc-Madison	Tree based (CART, BART, Random forest, gradient boosting, etc.), Ensemble	0.688	0.575	num_iterations, learning_rate, max_depth, min_data_in_leaf, lambda_l, lambda_l2
AI4knowledgeLAB	Tree based (CART, BART, Random forest, gradient boosting, etc.), Ensemble, Over-sampling of the minority class (SMOTE)	0.641	0.484	Random Forest(n_estimators=100, criterion='gini', max_depth=None, min_samples_leaf=1, min_samples_split=2, min_weight_fraction_leaf=0.0, max_features='sqrt')<br><br>SMOTE(sampling_strategy='auto')
IBB_UT	Tree based (CART, BART, Random forest, gradient boosting, etc.), Cluster based (k-means, spectral cluster, NMF, PCA)	0.64	0.526	ntreetry=500 for tuneRF function
KBJ	Ensemble	0.635	0.538	vanila sklearn models (default parameters)
DMIS_2022_PTB	Tree based (CART, BART, Random forest, gradient boosting, etc.), Ensemble	0.634	0.47	learning_rate, max_depth, n_estimators, alpha, lambda
yuanfang.guan	Tree based (CART, BART, Random forest, gradient boosting, etc.)	0.631	0.52	n_features
Team Wallac	Neural Net	0.629	0.563	-Modeling parameters:<br>Selection of predictors<br>- Neural network parameters:<br>Selection of hidden layers, type of normalization, amount of dropout, Selection of optimizer and loss function<br>- NN hidden layer parameters:<br>number of filters, kernel size, activation function
MatsuiLab	Regression (includes linear, logistic, CoxPH, Poisson, generalized, etc.), Tree based (CART, BART, Random forest, gradient boosting, etc.), Cluster based (k-means, spectral cluster, NMF, PCA)	0.625	0.515	NIH Racial Category, Age, Phylotypes, cst

UMICH_DREAM_PTB	Tree based (CART, BART, Random forest, gradient boosting, etc.)	0.623	0.54	n_estimators, max_depth, min_samples_split, criterion
Korem_group	Regression (includes linear, logistic, CoxPH, Poisson, generalized, etc.)	0.62	0.498	We tuned L1/L2 penalties, PCA components/kernels.
UTK-Bioinformatics_Preterm	Tree based (CART, BART, Random forest, gradient boosting, etc.), Support Vector Machine, Ensemble	0.62	0.487	n_features, min_leaf
IBSE_IITM	Tree based (CART, BART, Random forest, gradient boosting, etc.)	0.619	0.58	n_estimators, min_samples_split,min_samples_leaf,criterion
Clague VT.TP	Tree based (CART, BART, Random forest, gradient boosting, etc.)	0.605	0.523	all variables from microbial diversity, community state types, and phylotype relative abundance matrices.
Aagaard Lab - Baylor College of Medicine - Texas Childrens Hospital		0.604	0.514	
Techtmann Lab	Tree based (CART, BART, Random forest, gradient boosting, etc.), Neural Net	0.604	0.53	None other than default Scikit-learn v. 1.2.0 parameters for random forests (n_estimators = 100, criterion = gini)<br><br>Neural nets hyperparameters: learning rate = 1e-4, 3:1 = critic:generator training ratio, gradient penalty weight = 10, latent dim = 1000, batch size = 64, epochs = 10000
Drexel EESI	Neural Net	0.602	0.547	Some free hyperparameters in our modeling were the parameters used for our neural network, which included two intertwined autoencoder (ae) and discriminator (disc) components. The parameters for these were: nlayers_ae = 5; nnodes_ae = [1000,500, 250, 500, 1000], dropout_ae = 0.4, nlayers_disc = 3, nnodes_disc = [512, 256, 128], and dropout_disc = 0.2.

HOPWAS	Tree based (CART, BART, Random forest, gradient boosting, etc.), Cluster based (k-means, spectral cluster, NMF, PCA), Ensemble<br><br>CNN LSTM model	0.574	0.496	n_featurs, min_samples_leaf, max_depth, min_features_to_select<br><br>We use CNN LSTM deep learning model, and the weights of the designed network architecture are the parameters.

Supplementary Table 5: Summary of the approach and methods for the top-performing models for Sub-challenge 2 (early preterm birth). Related to STAR Methods and figure 3.

		**Performance Metrics**	**Performance Metrics**	
**Team**	**Modeling Methods**	**AUROC**	**AUPRC**	**Hyperparameters**
Techtmann Lab	Tree based (CART, BART, Random forest, gradient boosting, etc.)	0.868	0.446	None other than default Scikit-learn v. 1.2.0 parameters (n_estimators = 100, criterion = gini)
KBJ	Ensemble	0.841	0.27	vanila sklearn models
AI4knowledgeLAB	Tree based (CART, BART, Random forest, gradient boosting, etc.), Ensemble, Over-sampling of the minority class (SMOTE)	0.831	0.343	Random Forest(n_estimators=100, criterion='gini', max_depth=None, min_samples_leaf=1, min_samples_split=2, min_weight_fraction_leaf=0.0, max_features='sqrt')<br><br>SMOTE(sampling_strategy='auto')
polalawang	Regression (includes linear, logistic, CoxPH, Poisson, generalized, etc.), Tree based (CART, BART, Random forest, gradient boosting, etc.), Support Vector Machine, Ensemble	0.827	0.297	n_features, alpha
IBSE_IITM	Tree based (CART, BART, Random forest, gradient boosting, etc.)	0.817	0.273	n_estimators, min_samples_split,min_samples_leaf,criterion
UMICH_DREAM_PTB	Tree based (CART, BART, Random forest, gradient boosting, etc.)	0.816	0.442	n_estimators, max_depth, min_samples_split, criterion
Clague VT.TP	Tree based (CART, BART, Random forest, gradient boosting, etc.)	0.807	0.35	all variables in microbial diversity, community state types, and phylotype relative abundance matrices.
USF biostat	Tree based (CART, BART, Random forest, gradient boosting, etc.)	0.803	0.272	Phylotypes
yuanfang.guan	Tree based (CART, BART, Random forest, gradient boosting, etc.)	0.769	0.189	n_features

Supplementary Table 6: Feature importance across top-performing models and univariate association with preterm birth for sub-challenge 1. Related to Figure 4.

Available in CSV format.

Supplementary Table 7: Feature importance across top-performing models and univariate association with early preterm birth for sub-challenge 2. Related to Figure 4.

Available in CSV format

Supplementary Table 8: The Preterm Birth DREAM Community - consortium author affiliations. Related to Author List.

Yong Ju Ahn[1], Yadid M. Algavi[2], Nicola Amoroso[3,4], Maria De Angelis[5], George Austin[6,7], Ashley Babjac[8], Daehun Bae[9], Seungheun Baek[10], Roberto Bellotti[4,11], Panayiotis Benos[12], Yonatan Berg[2], Isaac Bigcraft[13], Aya Brown-Kav[6], Kun Bu[14], Guanhua Chen[15], Jhih-Yu Chen[16], Sz-Hau Chen[17], Tsai-Min Chen[18,19], Feng Cheng[20,21], Junseok Choe[10], Francesco Cremonesi[22], Saishi Cui[23], Yang Dai[24], Scott Emrich[8], Alonso Felipe-Ruiz[25], Diego Fernandez-Edreira[26,27], Carlos Fernandez-Lozano[26,27], Jifan Gao[15], Sergio Pérez García[28], Mogan Gim[10], Enrico Glaab[29], Akhil Goel[30], Ella Goldschmidt[31], Igor Goryanin[32,33], Yuanfang Guan[34], Dror Hadas[31], Kyudong Han[35], Weiru Han[14], Chih-Han Huang[36], Kuei-Lin Huang[37], Hirotaka Iijima[38,39], Gwanghoon Jang[10], Jongbum Jeon[40], Hongmei Jiang[41], Michael Jochum[42], Soobok Joe[40], Jaewoo Kang[10,43], Tina Khajeh[24], Eunyoung Kim[9], Hajung Kim[43], Jiwoong Kim[44,45], William Kindschuh[6], Stephanie Kivlin[46], Hayata Kodama[38], Aki Koivu[47], Tal Korem[6,48], Abigail Kuntzleman[13], Manuel E. González Lastre[49], Mo Li[50], Jose Linares-Blanco[51,52], Wodan Ling[53], Tyler C Lovelace[54,55], Jiuyao Lu[50], Zhixiu Lu[8], Jiangyue Mao[30], Miguel Pineda Martín[56], Yusuke Matsui[38,57], Kevin McPherson[58], Alfonso Monaco[4,11], Hesam Montazeri[59], Chengcheng Mou[60], Efrat Muller[31], Akiha Nakano[61], Saina Nassiri[62], Sina Nassiri[63], Naresh Nelaturi[64], Milad Norouzi[59], Pierfrancesco Novielli[4,5], Indumathi Palanikumar[65,66,67], Tanay Panja[68], Ester Pantaleo[4,11], Itsik Pe'er[6,7], Omri Peleg[31], Anna Plantinga[69], Augustinas Prusokas[58], Alisa Prusokiene[70], Karthik Raman[65,66,67], Derek Reiman[71], Renata Retkute[72], Donato Romano[4,5], Gail Rosen[73], Mohammad Sadegh Vafaei Sadi[59], Mikko Sairanen[47], Hibiki Sakata[38], Ricardo Paixao dos Santos[74], Edward S.C. Shih[75], Koji Shimazaki[38], Guy Shur[2], Alireza Fotuhi Siahpirani[59], Vijaya Yuvaram Singh V M[66,67,76], Himanshu Sinha[65,66,67], Bahrad Sokhansanj[73], Anatoly Sorokin[32], Go Suhara[61], Zheng-Zheng Tang[15], Sabina Tangaro[4,5], Victor Tarca[77], Stephen Techtmann[13], Manoj Teltumbade[64], Ambuj Tewari[30], Gabriel Trigo[7], Mor Tsamir[2], Kako Tsukioka[61], Kohei Uno[38], Mirco Vacca[5], Hsuan-Kai Wang[58], Huiqian Wang[30], Zehua Wang[30], Zijing Wang[78], Zidan Wang[41], Zhoujingpeng Wei[15], Chih-Hsun Wu[79], Michael C. Wu[80], Shaoming Xiao[81], Ryota Yanase[61], Jiaming Yao[30], Issa Zakeri[23], Wenjie Zeng[12], Xiaowei Zhan[44,45], Liangliang Zhang[82], Yuci Zhang[30], Ni Zhao[50]

[1]Division of Microbiome, HuNBiome Co., Ltd., Seoul, Republic of Korea.
[2]Sackler Faculty of Medicine, Tel Aviv University, Tel Aviv, Israel.
[3]Dipartimento di Farmacia - Scienze del Farmaco, Universita degli Studi di Bari A. Moro, Bari, Italy.
[4]Istituto Nazionale di Fisica Nucleare, Sezione di Bari, Bari, Italy.
[5]Dipartimento di Scienze del Suolo, della Pianta e degli Alimenti, Universita degli Studi di Bari A. Moro, Bari, Italy.
[6]Program for Mathematical Genomics, Department of Systems Biology, Columbia University Irving Medical Center, New York, New York, United States.
[7]Department of Computer Science, Columbia University, New York, New York, United States.
[8]Department of Computer Science, Univeristy of Tennessee, Knoxville, Tennessee, United States.
[9]School of Electrical Engineering and Computer Science, Gwangju Institute of Science and Technology, Gwangju, Republic of Korea.
[10]Department of Computer Science and Engineering, Korea University, Seoul , Republic of Korea.
[11]Dipartimento Interateneo di Fisica M. Merlin, Universita degli Studi di Bari A. Moro, Bari, Italy.
[12]Epidemiology, University of Florida College of Public Health and Health Professions and College of Medicine, Gainesville, Florida, United States.
[13]Department of Biological Sciences, Michigan Technological University, Houghton, Michigan, United States.
[14]Department of Mathematics & Statistics, College of Art and Science, University of South Florida, Tampa, Florida, United States.
[15]Department of Biostatistics and Medical Informatics, University of Wisconsin-Madison, Madison, Wisconsin, United States.
[16]Graduate Institute of Biomedical Electronics and Bioinformatics, National Taiwan University, Taipei, Taiwan.
[17]Industrial Information Department, Development Center for Biotechnology, Taipei, Taiwan.
[18]Graduate Program of Data Science, National Taiwan University and Academia Sinica, Taipei, Taiwan.
[19]Research Center for Information Technology Innovation, Academia Sinica, Taipei, Taiwan.
[20]Department of Pharmaceutical Science, Taneja College of Pharmacy, University of South Florida, Tampa, Florida, United States.
[21]Department of Biostatistics & Epidemiology, College of Public Health, University of South Florida, Tampa, Florida, United States.
[22]Centre Inria d'Université Côte d'Azur, Centre Inria, Biot, Sophia Antipolis, France.
[23]Department of Epidemiology and Biostatistics, Drexel University, Philadelphia, Pennsylvania, United States.
[24]Department of Biomedical Engineering, University of Illinois Chicago, Chicago, Illinois, United States.

[25]Department of Genomics and Proteomics, Institute of Biomedicine of Valencia, Valencia, Spain.
[26]Department of Computer Science and Information Technologies, Universidade da Coruña, A Coruña, Spain.
[27]CITIC-Research Center of Information and Communication Technologies, Universidade da Coruña, A Coruña, Spain.
[28]Data Engineer, Damavis Studio, Palma de Mallorca, Spain.
[29]Luxembourg Centre for Systems Biomedicine, University of Luxembourg, Esch-sur-Alzette, Luxembourg.
[30]Department of Statistics, University of Michigan, Ann Arbor, Michigan, United States.
[31]Blavatnik School of Computer Science, Tel Aviv University, Tel Aviv, Israel.
[32]Biological Systems Unit, Okinawa Institute of Science and Technology, Onna, Okinawa, Japan.
[33]School of Informatics, University of Edinburgh, Edinburgh, Midlothian, United Kingdom.
[34]Department of Computational Medicine and Bioinformatics, University of Michigan, Ann Arbor, Michigan, United States.
[35]Department of Microbiology, Dankook University, Cheonan, Republic of Korea.
[36]Department of Product, ANIWARE, Taipei, Taiwan.
[37]School of Medicine, China Medical University, Taichung, Taiwan.
[38]Graduate School of Medicine, Nagoya University, Nagoya, Aichi, Japan.
[39]Institute for Advanced Research, Nagoya University, Nagoya, Aichi, Japan.
[40]Korea BioInformation Center, Korea Research Institute of Bioscience & Biotechnology, Daejeon, Republic of Korea.
[41]Department of Statistics and Data Science, Northwestern University, Evanston, Illinois, United States.
[42]Obstetrics and Gynecology, Baylor College of Medicine and Texas Children's Hospital, Houston, Texas, United States.
[43]Interdisciplinary Graduate Program in Bioinformatics, Korea University, Seoul, Republic of Korea.
[44]Peter O'Donnell Jr. School of Public Health, University of Texas Southwestern Medical Center, Dallas, Texas, United States.
[45]Quantitative Biomedical Research Center, University of Texas Southwestern Medical Center, Dallas, Texas, United States.
[46]Department of Ecology, University of Tennessee, Knoxville, Tennessee, United States.
[47]Advanced Research and Technology, R&D PerkinElmer, Wallac Oy, Turku, Finland.
[48]Department of Obstetrics and Gynecology, Columbia University Irving Medical Center, New York, New York, United States.
[49]Department of Theoretical Condensed Matter Physics, Universidad Autónoma de Madrid, Madrid, Spain.
[50]Department of Biostatistics, Johns Hopkins University, Baltimore, Maryland, United States.
[51]Department of Statistics, University of Granada, Granada, Spain.
[52]GENYO. Centre for Genomics and Oncological Research: Pfizer, University of Granada, Granada, Spain.
[53]Biostatistics Division in the Population Health Sciences Department, Weill Cornell Medicine, New York, New York, United States.
[54]Department of Computational & Systems Biology, University of Pittsburgh, Pittsburgh, Pennsylvania, United States.
[55]Joint CMU-Pitt PhD Program in Computational Biology, Carnegie Mellon University and University of Pittsburgh, Pittsburgh, Pennsylvania, United States.
[56]Matematician, University of Seville, Seville, Spain.
[57]Institute for Glyco-core Research (iGCORE), Nagoya University, Nagoya, Aichi, Japan.
[58]Independent Researcher.
[59]Department of Bioinformatics, Institute of Biochemistry and Biophysics, University of Tehran, Tehran, Iran.
[60]Department of Computer Science and Engineering, College of Engineering, University of South Florida, Tampa, Florida, United States.
[61]School of Health Sciences, Nagoya University, Nagoya, Aichi, Japan.
[62]Department of Gynecology and Obstetrics, Tehran University of Medical Sciences, Tehran, Iran.
[63]Pharma Research and Early Development, Roche, Basel, Switzerland.
[64]CognitiveCare Inc, CognitiveCare Inc, Milpitas, California, United States.
[65]Department of Biotechnology, Bhupat and Jyoti Mehta School of Biosciences, Indian Institute of Technology Madras, Chennai, India.
[66]Centre for Integrative Biology and Systems Medicine, Indian Institute of Technology Madras, Chennai, India.
[67]Robert Bosch Centre for Data Science and Artificial Intelligence, Indian Institute of Technology Madras, Chennai, India.
[68]Huron High School, Huron High School, Ann Arbor, Michigan, United States.
[69]Department of Mathematics and Statistics, Williams College, Williamstown, Massachusetts, United States.
[70]School of Natural and Environmental Sciences, Newcastle University, Newcastle, United Kingdom.
[71]Toyota Technological Institute at Chicago, Toyota Technological Institute at Chicago, Chicago, Illinois, United States.
[72]Department of Plant Sciences, University of Cambridge, Cambridge, United Kingdom.
[73]Department of Electrical & Computer Engineering, Drexel University, Philadelphia, Pennsylvania, United States.
[74]Department of Medicine, Universidade de São Paulo, São Paulo, Brazil.
[75]Institute of Biomedical Sciences, Academia Sinica, Taipei, Taiwan.

[76]Department of Biotechnology, Indian Institute of Technology Madras, Chennai, India.
[77]Clague Middle School, Clague Middle School, Ann Arbor, Michigan, United States.
[78]Data Science Institute, Columbia University, New York, New York, United States.
[79]Artificial Intelligence and E-learning Center, National Chengchi University, Taipei, Taiwan.
[80]Public Health Sciences Division, Fred Hutchinson Cancer Research Center, Washington, Seattle, United States.
[81]School of Medicine, Johns Hopkins University, Baltimore, Maryland, United States.
[82]Department of Population and Quantitative Health Sciences, Case Western Reserve University, Cleveland, Ohio, United States.
